# The *Drosophila* Mi-2 Chromatin-Remodeling Factor Regulates Higher-Order Chromatin Structure and Cohesin Dynamics *In Vivo*

**DOI:** 10.1371/journal.pgen.1002878

**Published:** 2012-08-09

**Authors:** Barbara Fasulo, Renate Deuring, Magdalena Murawska, Maria Gause, Kristel M. Dorighi, Cheri A. Schaaf, Dale Dorsett, Alexander Brehm, John W. Tamkun

**Affiliations:** 1 Department of Molecular, Cell, and Developmental Biology, University of California Santa Cruz, Santa Cruz, California, United States of America; 2 Institute for Molecular Biology and Tumor Research (IMT), Philipps-University of Marburg, Marburg, Germany; 3 Edward A. Doisy Department of Biochemistry and Molecular Biology, Saint Louis University School of Medicine, Saint Louis, Missouri, United States of America; Massachusetts Institute of Technology, United States of America

## Abstract

dMi-2 is a highly conserved ATP-dependent chromatin-remodeling factor that regulates transcription and cell fates by altering the structure or positioning of nucleosomes. Here we report an unanticipated role for dMi-2 in the regulation of higher-order chromatin structure in *Drosophila*. Loss of dMi-2 function causes salivary gland polytene chromosomes to lose their characteristic banding pattern and appear more condensed than normal. Conversely, increased expression of dMi-2 triggers decondensation of polytene chromosomes accompanied by a significant increase in nuclear volume; this effect is relatively rapid and is dependent on the ATPase activity of dMi-2. Live analysis revealed that dMi-2 disrupts interactions between the aligned chromatids of salivary gland polytene chromosomes. dMi-2 and the cohesin complex are enriched at sites of active transcription; fluorescence-recovery after photobleaching (FRAP) assays showed that dMi-2 decreases stable association of cohesin with polytene chromosomes. These findings demonstrate that dMi-2 is an important regulator of both chromosome condensation and cohesin binding in interphase cells.

## Author Summary

The packaging of DNA into chromatin is critical for the organization and expression of eukaryotic genes. Nucleosomes repress transcription by blocking the access of transcription factors and other regulatory proteins to DNA. Levels of chromatin organization above the level of the nucleosome—including chromosome folding, pairing, and looping—can also have profound effects on gene expression. However, the mechanisms by which higher-order chromatin structure is regulated and used to control gene expression remain poorly understood. Using *Drosophila* as a model organism, we have discovered an unanticipated role for dMi-2, a well-characterized ATP-dependent chromatin- remodeling factor, in the regulation of higher-order chromatin structure and cohesin dynamics *in vivo*. The inhibition of dMi-2 function causes chromosomes to condense, while elevated expression of dMi-2 triggers the decondensation of polytene and mitotic chromosomes and also destabilizes cohesin binding. Our findings suggest that dMi-2 may regulate transcription and cellular differentiation in other organisms, including humans, by altering higher-order chromatin structure or cohesin dynamics.

## Introduction

The packaging of DNA into chromatin is critical for the organization and expression of eukaryotic genes [1], [2], [3]. The basic unit of chromatin structure, the nucleosome, contains the core histones H2A, H2B, H3 and H4. The association of nucleosomes with histone H1 and other linker histones facilitates their packaging into 30 nm fibers, which in turn are packaged into increasingly compact higher-order structures. Nucleosomes and other components of chromatin can repress transcription by blocking the access of regulatory proteins and the basal transcriptional machinery to DNA. There is growing evidence that levels of chromosome organization above the level of the nucleosome – including chromosome folding, pairing and looping – also play important roles in the regulation of gene expression. For example, condensin and cohesin, which were initially identified by their roles in mitosis and meiosis, modulate transcription by promoting long-range chromosomal interactions and DNA looping in interphase cells [4].

The repressive effects of nucleosomes on transcription are modulated by two general mechanisms: the covalent modification of nucleosomal histones and ATP-dependent chromatin remodeling [1], [5]. By altering the structure or positioning of nucleosomes, ATP-dependent chromatin-remodeling factors play critical roles in transcription and other nuclear processes. Dozens of chromatin-remodeling factors, including members of the SWI/SNF, ISWI, CHD and INO80 families, have been identified in organisms ranging from yeast to humans. By contrast, relatively little is known about how higher-order chromatin structure is regulated and exploited to control gene expression and other nuclear processes.

A major barrier to the identification of factors that regulate higher-order chromatin structure is the difficulty of visualizing the decondensed interphase chromosomes of diploid cells. This barrier can be overcome through the use of *Drosophila melanogaster* as a model organism. During *Drosophila* development, many tissues undergo multiple rounds of DNA replication in the absence of cytokinesis, leading to the formation of huge polytene chromosomes containing hundreds of aligned sister chromatids. These transcriptionally active chromosomes are indistinguishable from the interphase chromosomes of diploid cells in most respects. Genetic studies in *Drosophila* have identified numerous factors that regulate polytene chromosome structure, including ISWI, an ATP-dependent chromatin-remodeling factor. The loss of *ISWI* function leads to the decondensation of salivary gland polytene chromosomes, possibly due to failure to assemble chromatin containing the linker histone H1 [6], [7], [8]. This striking phenotype led us to investigate the potential involvement of another ATP-dependent chromatin-remodeling factor, *Drosophila* Mi-2 (dMi-2), in the regulation of higher-order chromatin structure.

dMi-2 functions as the ATPase subunit of multiple chromatin-remodeling complexes, including the NuRD (Nucleosome Remodeling and Deacetylase) complex and dMec (*Drosophila* MEP-1 containing complex) [9]. NuRD is highly conserved in metazoans and is thought to repress transcription via its chromatin-remodeling and histone deacetylase activities [10], [11], [12]. dMec is the most abundant dMi-2 complex in *Drosophila* and has been implicated in SUMO-dependent transcriptional repression [13], [14]. Mi-2 plays an important role in cell fate specification in organisms ranging from nematodes to vertebrates. For example, Mi-2 helps maintain the distinction between the germline and soma during *C. elegans* embryogenesis [15]; regulates the terminal differentiation of B lymphocytes into plasma cells in mammals [16]; and participates in the transcriptional repression of HOX genes by Hunchback and Polycomb in *Drosophila* [17]. dMi-2 is also required for the efficient expression of heat-shock genes in *Drosophila*, indicating that its function is not limited to transcriptional repression [18].

Here we report an unanticipated role for Mi-2 in the regulation of higher-order chromatin structure in *Drosophila*. The loss of dMi-2 function causes salivary gland polytene chromosomes to lose their characteristic banding pattern and appear more condensed than normal. Conversely, the increased expression of dMi-2 in the salivary gland disrupts interactions between sister chromatids and triggers the decondensation of polytene chromosomes. Consistent with these findings, dMi-2 disrupts the association of cohesin with polytene chromosomes. Our studies reveal that dMi-2 is an important regulator of both chromosome condensation and cohesin binding in interphase cells.

## Results

### Generation and analysis of GAL4-regulated transgenes encoding wild-type and dominant-negative dMi-2 proteins

As a first step toward investigating the role of dMi-2 in the regulation of higher-order chromatin structure, we investigated phenotypes resulting from either the loss or gain of dMi-2 function. We could not examine the polytene chromosomes of individuals homozygous for loss-of-function *dMi-2* alleles since they die relatively early in larval development [17], [19]. We therefore used the GAL4 system [20] to examine the consequences of expressing a dominant-negative form of the dMi-2 protein in the salivary gland. For this study, we used a mutation that deletes amino acids 932-1158 of the dMi-2 protein, including its bipartite ATP-binding site. This deletion eliminates the ATPase activity of dMi-2 *in vitro* [21] without disrupting its interaction with other proteins (Figure S1). We therefore reasoned that the expression of dMi-2^Δ932-1158^ should have a strong, dominant-negative effect on dMi-2 function *in vivo*, as has been observed for comparable mutations in other chromatin-remodeling factors [7], [22], [23].

Transgenic fly strains bearing GAL4-inducible transgenes encoding wild-type (*UAS-dMi-2^+^*) or dominant-negative (*UAS-dMi-2^Δ932-1158^*) dMi-2 proteins were generated by P element transformation. Transformants were crossed to GAL4 driver lines to generate progeny that express the *dMi-2* transgenes in stage or tissue-specific patterns. The GAL4 system is inherently temperature-sensitive; higher levels of transgene expression are observed at higher temperatures [24]. This allowed us to modulate the expression of *dMi-2* transgenes by varying the temperature at which progeny were reared.

The expression of *dMi-2^Δ932-1158^* transgenes under the control of a ubiquitously expressed GAL4 driver (*da-GAL4*) had a dramatic effect on viability. At 25°C, the majority of *UAS-dMi-2^Δ932-1158^ 6-5/+; da-GAL4/+* individuals completed larval development, but only 5% survived to adulthood (Table 1). The lethality resulting from the expression of dMi-2^Δ932-1158^ was moderately enhanced by a hypomorphic allele of dMi-2 (*dMi-2^f08103^*) and strongly enhanced by the null allele *dMi-2^4^* (Table 1). At 29°C, no individuals expressing dMi-2^Δ932-1158^ under the control of the *da-GAL4* driver survived beyond the first or second larval instar (data not shown). This lethal phase is identical to that resulting from the complete loss of zygotic dMi-2 function [17], [19]. These findings confirmed that the expression of the catalytically inactive dMi-2^Δ932-1158^ protein has strong, dominant-negative effects on dMi-2 function *in vivo*.

**Table 1:** Analysis of GAL4-regulated transgenes.

Genotype	number of copies			% survival to	
	dMi-2^+^	UAS-dMi-2^Δ932-1158^	UAS-dMi-2^+^	pupae	adults
*dMi-2^4^/da-GAL4* (a)	1	0	0	93.9	92.8
*dMi-2^4^/dMi-2^4^ da-GAL4* (b)	0	0	0	0.0	0.0
*UAS-dMi-2* ^Δ*932-1158*^ 6-5/+; da-GAL4/+(c)	2	1	0	74.0	5.0
*UAS-dMi-2* ^Δ*932-1158*^ 6-5/+; dMi-2^f08103^/da-GAL4(d)	1	1	0	23.0	9.6
*UAS-dMi-2* ^Δ*932-1158*^ 6-5/+; dMi-2^4^/da-GAL4(e)	1	1	0	0.0	0.0
*UAS-dMi-2^+^ 3-3/+; dMi-2^4^/dMi-2^4^ da-GAL4* (f)	0	0	1	76.0	73.9
*UAS-dMi-2^+^ 3-3/+; da-GAL4/+* (g)	2	0	1	13.9	12.1
*UAS-dMi-2^+^ 3-3/+; dMi-2^4^/da-GAL4* (h)	1	0	1	88.2	82.6

Data are shown for progeny of the following crosses:

(a) *dMi-2^4^/TM6B*, *Tb* males X *w; da-GAL4* females;

(b) *w; dMi-2^4^/TM6B*, *Tb* males X *w; dMi-2^4^ da-GAL4/TM6B*, *Tb* females;

(c) *w; P[w^+^, UAS-dMi-2* ^Δ*932-1158*^]6-5 males X *w; da-GAL4* females;

(d) *w; P[w^+^, UAS-dMi-2* ^Δ*932-1158*^]6-5; dMi-2^f08103^/TM6B, *Tb* males X *w; da-GAL4* females;

(e) *w; P[w+, UAS-dMi-2* ^Δ*932-1158*^]6-5; dMi-2^4^/TM6B, *Tb* males X *w; da-GAL4* females;

(f) *w; P[w^+^, UAS-dMi-2^+^]3-3; dMi-2^4^/TM6B*, *Tb* males X *w; dMi-2^4^ da-GAL4/TM6B*, *Tb* females;

(g) *w; P[w^+^, UAS-dMi-2^+^]3-3* males X *w; da-GAL4* females;

(h) *w; P[w^+^, UAS-dMi-2^+^]3-3; dMi-2^4^/TM6B*, *Tb* males X *w; da-GAL4* females.

We also investigated the effect of expressing wild-type dMi-2 protein on *Drosophila* development using the *da-GAL4* driver. As expected, the *UAS-dMi-2^+^* transgene rescued the recessive lethality of *dMi-2^4^*; 74% of *UAS-dMi-2^+^ 3-3/+; dMi-2^4^ da-GAL4/dMi-2^4^* survived to adulthood (Table 1). Surprisingly, the over-expression of dMi-2^+^ in a wild-type dMi-2 background resulted in larval lethality; only 12% of *UAS-dMi-2^+^ 3-3/+; da-GAL4/+* individuals survived beyond the third-larval instar (Table 1). The lethality resulting from the expression of the *UAS-dMi-2^+^* transgene was suppressed by a twofold reduction in the level of endogenous dMi-2 function; over 80% of *UAS-dMi-2^+^ 3-3/+; dMi-2^4^/da-GAL4* individuals survived to adulthood (Table 1). Thus, even a modest increase in the level of dMi-2 can be lethal.

### Analysis of chromosome defects resulting from dMi-2 over-expression

**Figure 1:**
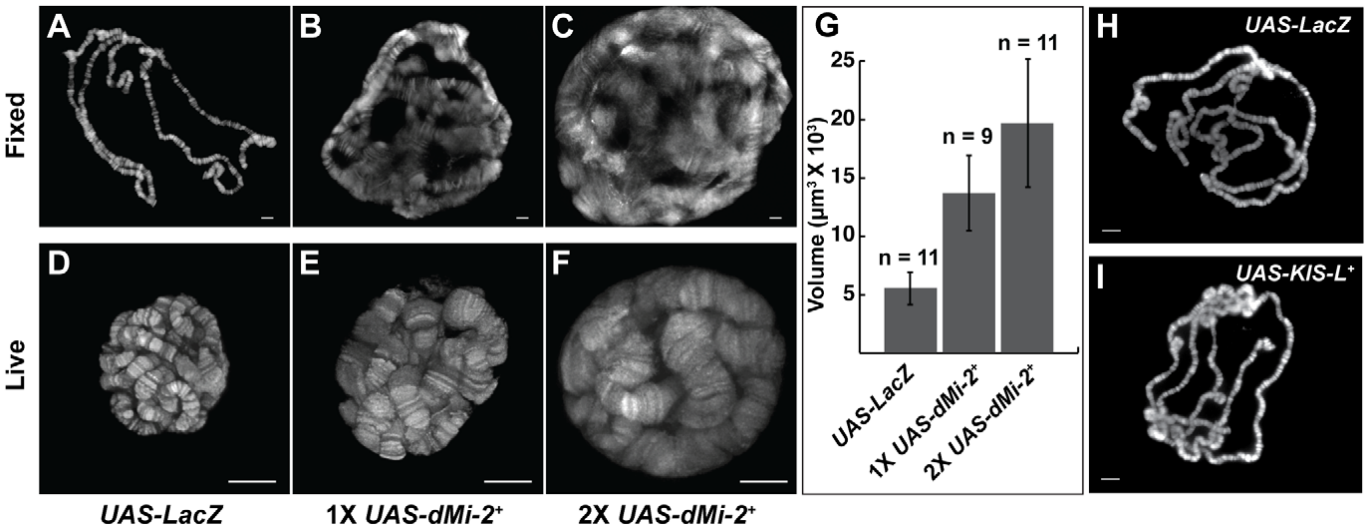
dMi-2 increases the size of polytene chromosomes *in vivo*. (A–F) Salivary gland polytene chromosomes of late third-instar *UAS-LacZ/+; da-GAL4/+* control larvae (A and D) or larvae bearing one (*UAS-dMi-2^+^ 3-3/+; da-GAL4/+* [1X *UAS-dMi-2^+^*]) (B and E) or two (*UAS-dMi-2^+^ 3-3/+*; *UAS-dMi-2^+^ 15-1/da-GAL4* (2X *UAS-dMi-2^+^*)] (C and F) copies of a *UAS-dMi-2^+^* transgene. (A–C) Squashes of fixed polytene chromosomes stained with DAPI. (D–F) Live analysis of chromosomes of larvae expressing His2Av-GFP. The over-expression of dMi-2 increases the size of polytene chromosomes (compare B and C to A and E and F to D) and compromises their structural integrity, as evidenced by the disruption of the banding pattern (compare B and C to A) (G) Quantification of the volume of polytene chromosomes of control, 1X *UAS-dMi-2^+^*, and 2X *UAS-dMi-2^+^* larvae imaged by live analysis. The over-expression of dMi-2 protein causes a two to fourfold increase in chromosome volume (1X *UAS-dMi-2^+^*: 1.4E+04±3.3E+03 µm^3^ and 2X *UAS-dMi-2^+^*: 1.97E+04±5.5E+03 µm^3^) compared to *UAS-Lac-Z* (5.6E+03±1.4E+03 µm^3^). (H–I) Over-expression of KIS-L has no obvious effect on the structure of polytene chromosomes. DAPI staining of polytene chromosomes squashes from *UAS-LacZ/ey-GAL4* (H) and *UAS-KIS-L^+^ 20-7/ey-GAL4* (I) individuals. A, B, C, D, E F, H and I scale bars are 10 µm. Larvae were reared at 29°C.

The above findings demonstrated that the GAL4 system can be used to analyze phenotypes resulting from either the gain or loss of dMi-2 function. We therefore proceeded to characterize chromosome defects resulting from the expression of wild-type or dominant-negative dMi-2 proteins. We initially used the *da-GAL4* driver to induce the expression of *UAS-dMi-2^Δ932-1158^* and *UAS-dMi-2^+^* transgenes in salivary gland nuclei. The increased expression of wild-type dMi-2 protein dramatically altered the structure of salivary gland polytene chromosomes; fixed polytene chromosomes squashes appeared much larger than normal; their banding pattern was severely disrupted; and it was often difficult to distinguish individual chromosome arms (compare Figure 1A and 1B). This phenotype was never observed in wild-type larvae (data not shown) or *UAS-LacZ/+; da-GAL4/+* control larvae (Figure 1A). Chromosome defects were even more pronounced in larvae bearing two copies of the *UAS-dMi-2^+^* transgene in addition to the *da-GAL4* driver (Figure 1C). Similar results were obtained with a *Sgs3-GAL4* driver which is expressed only in the salivary gland of third-instar larvae (Figure S2).

To gain a more accurate impression of the effect of increased dMi-2 expression on chromosome structure, we used live analysis to visualize the chromosomes of larvae expressing a fluorescently tagged core histone (His2Av-GFP). The salivary gland chromosomes of third-instar larvae expressing one or two *UAS-dMi-2^+^* transgenes under the control of the *da-GAL4* driver displayed a clear banding pattern but were much larger than normal (compare Figure 1D to 1E and 1F). The increased expression of dMi-2 led to a two to fourfold increase in chromosome volume (Figure 1G). The severe disruption of the chromosome banding pattern evident in fixed polytene chromosome squashes was not observed by live analysis (compare Figures 1B and 1C to 1E and 1F). This difference suggests the over-expression of dMi-2 makes chromosomes unusually sensitive to mechanical stress, leading to their disruption during the squashing procedure. Taken together, these observations suggest that the elevated expression of dMi-2 increases the size of polytene chromosomes while compromising their structural integrity.

### The analysis of chromosome defects resulting from the loss of dMi-2 function

**Figure 2:**
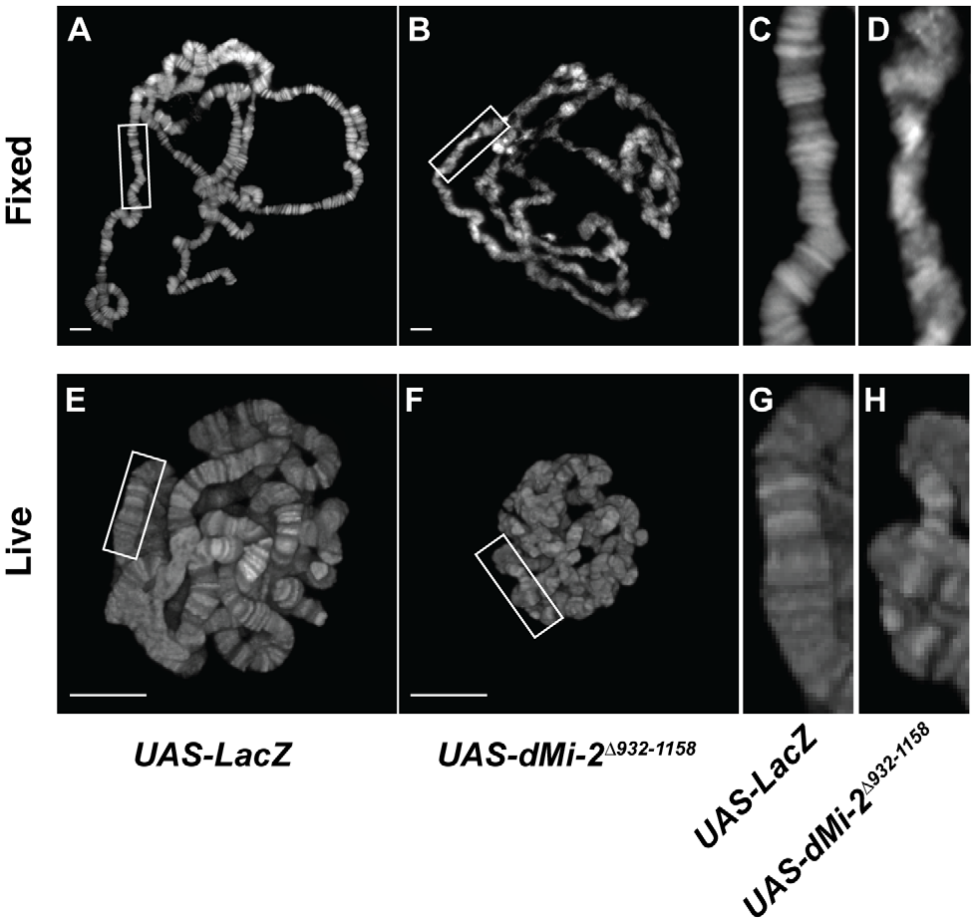
The loss of dMi-2 function alters chromosome structure. Salivary gland polytene chromosomes of late third-instar *UAS-LacZ/+; da-GAL4/+* control larvae (A and C) or larvae expressing the dominant-negative dMi-2^Δ*932-1158*^ protein (*UAS-dMi-2* ^Δ*932-1158*^ 6-5/+; da-GAL4/+) (B and D). (A–D) Squashes of fixed polytene chromosomes stained with DAPI. (E–H) Live analysis of chromosomes of larvae expressing His2Av-GFP. The expression of *dMi-2^Δ932-1158^ reduces the size of polytene chromosomes and disrupts their banding pattern. (Compare C and D to A and B, and G and H to E and F). A, B, E and F scale bars are 10 µm.*

We next examined the consequences of expressing the dominant-negative dMi-2^Δ932-1158^ protein in salivary gland nuclei. A significant fraction of *UAS-dMi-2^Δ932-1158^ 6-5/+; da-GAL4/+* individuals survive to adulthood at 25°C; at 29°C, they do not survive beyond the first or second larval instar (Table 1 and data not shown). To reduce dMi-2 function without blocking larval development, individuals of this genotype were shifted from 25°C to 29°C before the end of the first larval instar (0–48 hours after egg laying). Under these conditions, the loss of dMi-2 function disrupted the banding pattern of salivary gland chromosomes and caused them to appear much thinner than normal (compare Figure 2B and 2D to 2A and 2C). Similar results were obtained using a GAL4-regulated transgene encoding a catalytically inactive form of the dMi-2 protein (dMi-2^K761R^) in which a conserved lysine in the ATP-binding site is replaced with an arginine (MM and AB, unpublished data). Live analysis revealed chromosome defects similar to those observed in fixed chromosome squashes (compare Figure 2F and 2H to 2E and 2G). The ability of dMi-2 to increase the size of salivary gland polytene chromosomes is therefore dependent on its ATPase activity.

### The CHD protein KIS-L does not regulate higher-order chromatin structure *in vivo*

*Drosophila kismet* (*KIS*) was identified in genetic screens for *Polycomb* antagonists and subsequently shown to encode a member of the CHD subfamily of chromatin-remodeling factors [25], [26], [27]. The chromosomal distributions of KIS-L and dMi-2 are highly overlapping; both proteins are enriched at less condensed, transcriptionally active regions of chromatin (Figure S5A) [28]. These similarities prompted us to investigate whether the over-expression of KIS-L also alters the structure of salivary gland polytene chromosomes. Transgenic fly strains bearing GAL4-inducible transgenes encoding wild-type KIS-L protein, *UAS-KIS-L^+^*, were generated by P element transformation; the *da-GAL4* driver was used to drive the ubiquitous expression of these transgenes. As observed for dMi-2, the over-expression of the wild-type KIS-L protein is lethal; *UAS-KIS-L^+^ 20-7/+; da-GAL4/+* individuals failed to survive beyond late larval or early pupal stages of development (data not shown). Unlike dMi-2, however, the increased expression of KIS-L did not cause obvious changes in the structure of salivary gland polytene chromosomes (Figure 1H and 1I). The ability of dMi-2 to alter chromosome structure therefore does not appear to be a property common to all CHD proteins.

### The increased expression of wild-type dMi-2 triggers relatively rapid changes in chromosome structure

The chromosome defects described above resulted from the continuous expression of wild-type or dominant-negative dMi-2 proteins over a five to seven day period. To help distinguish between primary and secondary consequences of dMi-2 over-expression, we used a modification of the GAL4 system that permits the precise temporal regulation of GAL4-responsive transgenes through the use of a temperature-sensitive GAL80 repressor (GAL80^ts^) [29]. At the permissive temperature (18°C), GAL80^ts^ binds to and inhibits the GAL4 activator. At the restrictive temperature (29°C), GAL4 function is restored, leading to the rapid activation of the GAL4-responsive target gene. The use of this system allowed us to monitor the time course of changes in chromosome structure resulting from increased dMi-2 expression.

**Figure 3:**
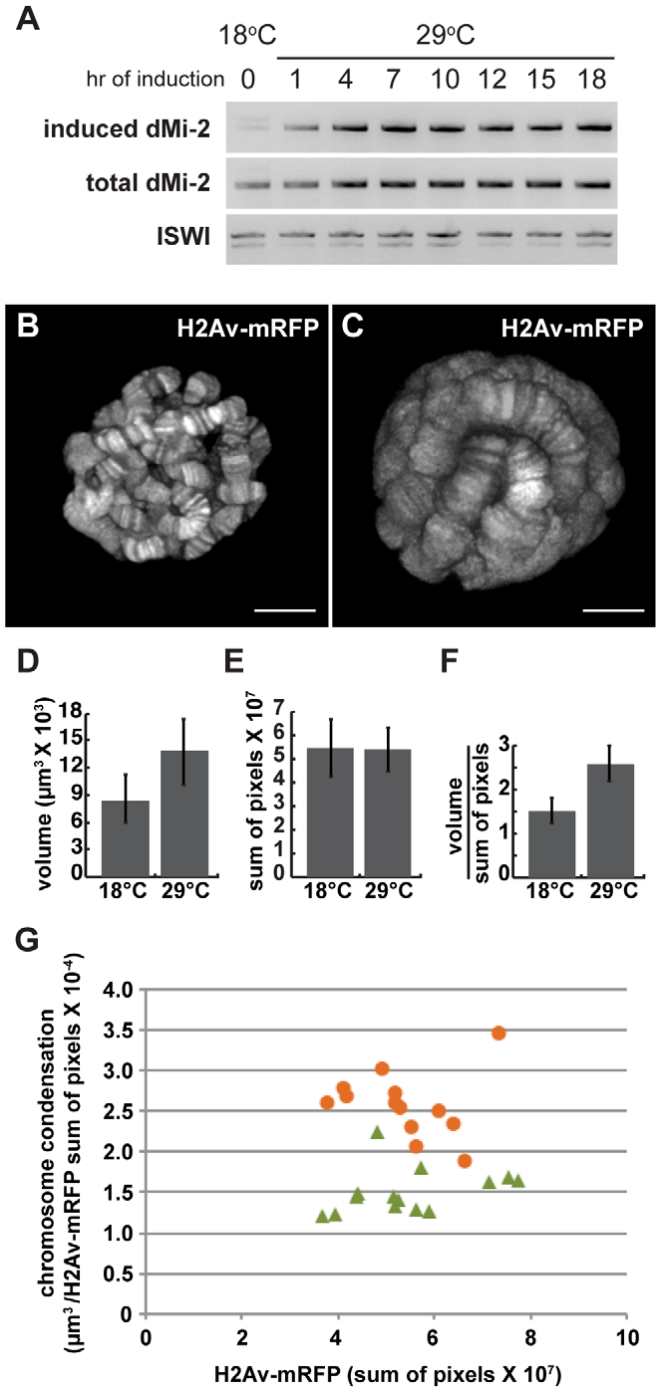
dMi-2 triggers rapid changes in chromosome structure without altering DNA replication. Third-instar *UAS-dMi-2^+^ 3-3/+; UAS-dMi-2^+^ 15-1/da-GAL4, Gal80^ts^* larvae were shifted from 18°C to 29°C to activate *UAS-dMi-2^+^* expression. (A) Changes in *dMi-2* RNA levels were monitored by RT-PCR following the shift from 18° to 29°C using primers that specifically amplify the RNA encoded by *UAS-dMi-2^+^* (induced) or both the induced and endogenous *dMi-2* RNAs (induced+endogenous). The level of ISWI RNA was assayed as an internal control. (B and C) Live analyses of salivary gland nuclei of *UAS-dMi-2^+^ 3-3/+*; *UAS-dMi-2^+^ 15-1/da-GAL4 GAL80^ts^* maintained at 18°C (B; n = 13) or shifted to 29°C for 27 hours (C; n = 14). (D) Quantification of the volume of salivary gland polytene chromosomes of larvae shown in B and C. (E) Quantification of His2Av-mRFP1 fluorescence in the same larvae shown in B and C. (F) Quantification of chromosome compaction, as assessed by the ratio of volume in µm^3^ to His2Av-mRFP1 fluorescence per nucleus for the larvae shown in B and C. (G) Scatter plot of the data used to generate D–F. Chromatin compaction vs. DNA content are compared for single nuclei of larvae maintained at 18°C (green triangles) or 29°C (orange circles) for 27 hours. B and C scale bars are 10 µm.

*UAS-dMi-2^+^ 3-3/+; UAS-dMi-2^+^ 15-1/da-GAL4 tubP-Gal80^ts^* individuals were shifted from 18°C to 29°C at the middle of the third larval instar to activate *UAS-dMi-2^+^* expression. RT-PCR was used to monitor the expression of the endogenous *dMi-2^+^* gene, the *UAS-dMi-2^+^* transgenes and the overall level of dMi-2 RNA during the subsequent 24 hours. The expression of the *UAS*-*dMi-2^+^* transgenes, but not the endogenous *dMi-2* gene, was rapidly activated after the shift to the restrictive temperature, leading to roughly a twofold increase in the total level of dMi-2 RNA within seven hours (Figure 3A). The level of dMi-2 RNA remained constant for the remainder of the experiment. Changes in chromosome structure were evident in squashed polytene chromosomes within ten to twelve hours after the shift to 29°C and became increasingly pronounced over the following twelve hours (Figure S3). The relatively short lag time between the increase in dMi-2 expression and alterations in chromosome morphology suggests that dMi-2 may directly regulate higher-order chromatin structure.

### dMi-2 does not increase the size of polytene chromosomes by altering DNA replication

Salivary gland polytene chromosomes consist of more than a thousand closely aligned sister chromatids formed by repeated rounds of DNA replication in the absence of cytokinesis. Additional rounds of DNA replication could therefore account for the huge size of salivary gland polytene chromosomes in larvae that express increased levels of dMi-2. To address this possibility, the above experiments were repeated using larvae expressing a core histone tagged with RFP (His2Av-mRFP1); this allowed us to use live analysis to directly compare changes in chromosome volume, DNA content (as estimated by the level of His2Av-mRFP1 fluorescence) and chromosome compaction (the ratio of chromosome volume to DNA content) resulting from increased dMi-2 expression. *UAS-dMi-2^+^ 3-3/His2Av-mRFP1; UAS-dMi-2^+^ 15-1/da-GAL4 tubP-Gal80^ts^* individuals were shifted from 18°C to 29°C to activate *UAS-dMi-2^+^* expression late in the third larval instar. 27 hours after the shift to 29°C, live analysis revealed a significant increase in polytene chromosome volume, but not His2Av-mRFP1 fluorescence, compared to larvae that had been maintained at 18°C, resulting in almost a twofold decrease in chromosome compaction (Figure 3B–G). Similar results were obtained when we compared the area of salivary gland polytene chromosome squashes to DNA content as estimated by DAPI fluorescence (data not shown). Increased dMi-2 expression thus triggers the decondensation of polytene chromosomes as opposed to additional rounds of replication.

**Figure 4:**
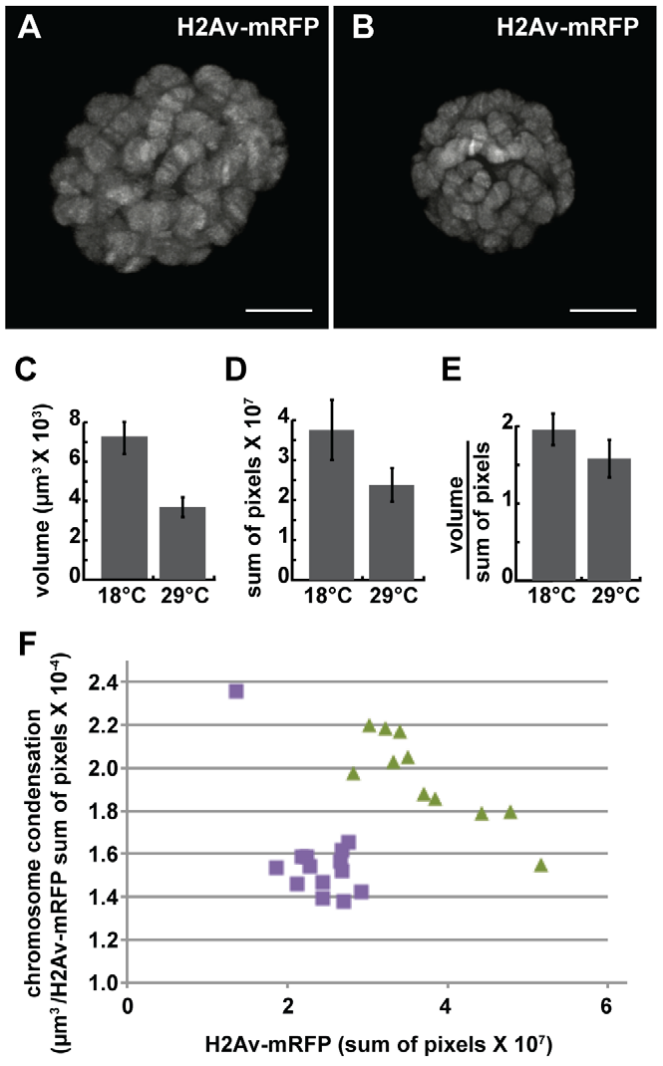
The loss of dMi-2 function promotes chromosome compaction. Third-instar *UAS-dMi-2* ^Δ*932-1158*^ 6-5/His2Av-mRFP1; +/da-GAL4 tubP-Gal80^ts^ larvae were shifted from 18°C to 29°C to activate *dMi-2* ^Δ*932-1158*^ expression. (A and B) Live analyses of salivary gland nuclei of *UAS-dMi-2* ^Δ*932-1158*^ 6-5/His2Av-mRFP1; +/da-GAL4 tubP-Gal80^ts^ larvae maintained at 18°C (A; n = 11) or shifted to 29°C for 48 hours (B; n = 14). (C–E) Quantification of the volume (C), His2Av-mRFP1 fluorescence (D), and compaction (E) of salivary gland chromosomes of larvae maintained at 18°C or 29°C for 48 hours after the temperature shift. (F) Scatter plot of the data used to generate C–E. Chromatin compaction vs. DNA content are compared for single nuclei of larvae maintained at 18°C (green triangles) or 29°C (purple circles) for 48 hours. A and B scale bars are 10 µm.

We next examined the effect of the dominant-negative *dMi-2* ^Δ*932-1158*^ protein on chromosome compaction using the GAL4-GAL80^ts^ system. *UAS-dMi-2* ^Δ*932-1158*^ 6-5/His2Av-mRFP1; +/da-GAL4 tubP-Gal80^ts^ individuals were shifted from 18°C to 29°C to activate *dMi-2* ^Δ*932-1158*^ expression late in the third larval instar. 48 hours after the shift to 29°C, live analysis revealed significant decreases in both polytene chromosome volume and His2Av-mRFP1 fluorescence relative to larvae maintained at 18°C (Figure 4A–D). A modest increase in chromosome compaction was also observed (Figure 4E); this effect was relatively striking when nuclei with similar DNA content were compared (Figure 4F). These findings provide additional evidence that dMi-2 is an important regulator of chromosome compaction *in vivo*.

### Loss of dMi-2 function reduces the size of heat-shock puffs

**Figure 5:**
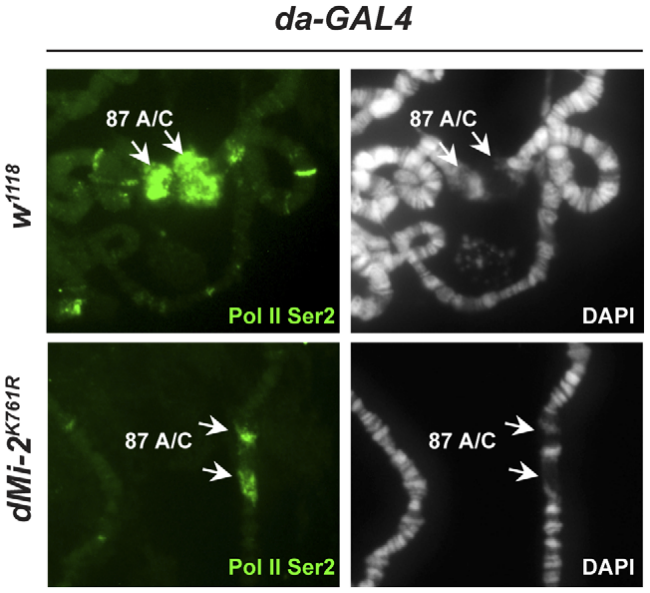
dMi-2 is required for full decondensation of heat-shock loci. *w^1118^* control and transgenic larvae expressing dominant-negative dMi-2^K761R^ were subjected to a 20 min heat-shock at 37°C. Polytene chromosomes were visualized by staining with DAPI (right panels) or indirect immunofluorescence using an antibody against Pol II Ser2 (left panels). Arrows indicate the *hsp70* gene containing loci 87A and 87C.

Activation of the *hsp70* heat-shock (HS) genes is accompanied by the formation of transcriptionally active HS puffs at loci 87A and 87C, a process that is generally attributed to chromosome decondensation. dMi-2 is recruited to HS genes following their activation [18]. We asked if loss of dMi-2 function would interfere with HS-induced chromosome puffing. We subjected third-instar larvae to a 20 minute HS at 37°C and visualized puff formation at 87A and 87C by indirect immunofluorescence using a RNA Pol II antibody. Under these conditions, all the polytene chromosomes of control larvae exhibited strong (67%) to moderate (33%) decondensation at 87A and 87C as judged by a loss of DAPI signal and an accumulation of RNA polymerase II (Figure 5, upper panels). By contrast, the extent of puff formation and the amount of polymerase II signal was significantly reduced in transgenic larvae expressing catalytically inactive dMi-2 (dMi-2^K761R^, raised at 25°C prior to heat-shock; Figure 5, lower panels); no puffs or only moderate puffing was observed in 14 and 53% of the chromosomes of these larvae, respectively. This result suggests that dMi-2 function is important for a local and rapid chromosome decondensation event.

### dMi-2 does not regulate higher-order chromatin structure by altering histone H1 assembly

**Figure 6:**
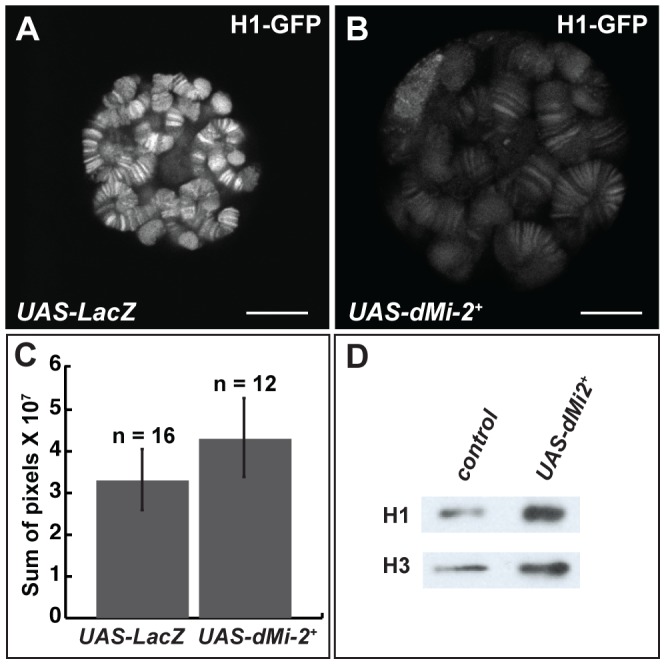
dMi-2 does not promote chromatin decondensation by antagonizing histone H1 assembly. (A–B) Live analysis of salivary gland nuclei of late third-instar *UAS-LacZ/+; ey-GAL4/+* (UAS-LacZ) control larvae (A) and *UAS-dMi-2^+^ 3-3/+*; *UAS-dMi-2^+^ 15-1/ey-GAL4* (*UAS-dMi-2^+^*) larvae (B) expressing H1-GFP. Scale bars are 10 µm. (C) Quantification of H1-GFP fluorescence in larvae shown in A and B. The exposure times used to capture the images are identical; the number of glands analyzed is noted. (D) Protein blot showing the relative levels of histones H1 and H3 in chromatin extracted from late third-instar *UAS-dMi-2^+^ 3-3/+*; *da-GAL4/+* (*UAS-dMi-2^+^*) and *UAS-LacZ/+*; *da-GAL4/+* (*UAS-LacZ*) larvae raised at 29°C.

The reduced expression of histone H1, a linker histone that promotes the formation of 30 nm fibers, causes polytene chromosome defects that are remarkably similar to those resulting from the increased expression of dMi-2 [8], [30]. This similarity suggested that dMi-2 might regulate chromosome structure by antagonizing the assembly of chromatin containing histone H1. To investigate this possibility, we examined whether the over-expression of dMi-2 decreases the association of histone H1 with chromatin. The live analysis of larvae expressing GFP-tagged histone H1 revealed that the elevated expression of dMi-2 increased the size of polytene chromosomes relative to control larvae without reducing the levels of bound histone H1 (Figure 6A–C). The over-expression of dMi-2 also did not reduce ISWI expression or the level of histone H1 in chromatin extracted from salivary glands as assayed by Western blotting (Figure 6D; Figure S4). The expression of dominant-negative dMi-2 in salivary gland nuclei caused a slight decrease in histone H1 levels (Figure S4). Thus, dMi-2 does not promote chromosome decondensation by repressing histone H1 expression or assembly.

### dMi-2 regulates interactions between sister chromatids in larval salivary gland polytene chromosomes

**Figure 7:**
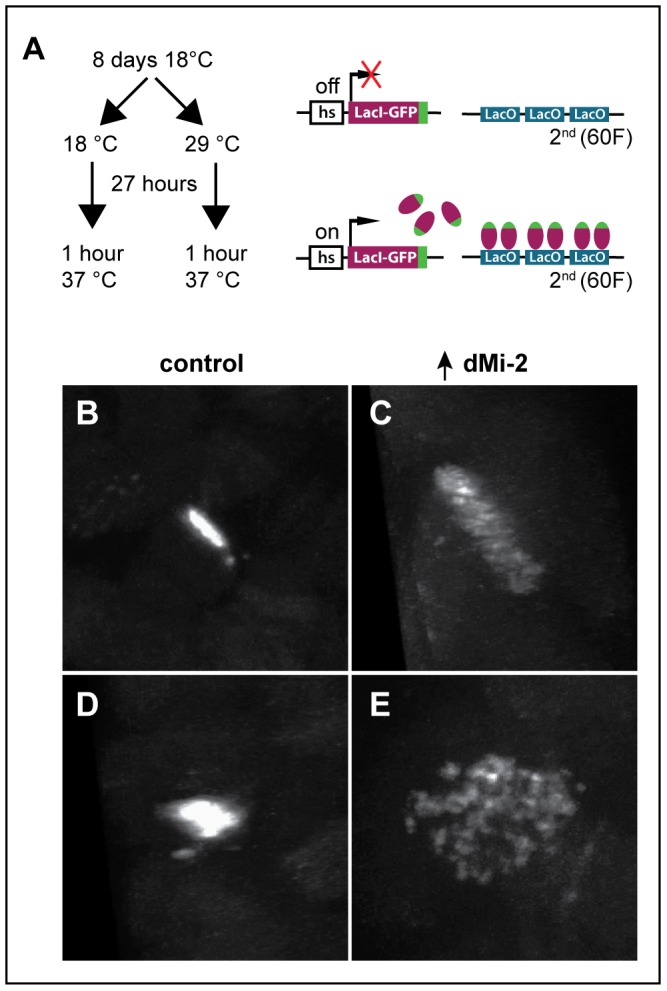
dMi-2 regulates chromosome cohesion in the larval salivary gland. (A) Overview of LacI-GFP/LacO tethering assay. The GFP-tagged LacI fusion protein (LacI-GFP) binds 256 tandem LacO sequences inserted at position 60F on the second chromosome allowing the detection of the locus in living cells. The *HS83* heat-shock promoter (hs) that drives *LacI-GFP* is activated at 37°C. (B-E) Live analysis of the LacI-GFP signal at 60F in the polytene chromosomes of third-instar larvae. *UAS-dMi-2^+^ 3-3/HS83-LacI-GFP LacO*; *UAS-dMi-2^+^ 15-1/da-GAL4 GAL80^ts^* individuals were cultured at 18°C until the middle of the third larval instar, maintained at 18°C (B and D) or shifted to 29°C (C and E) for 27 hours, and heat-shocked at 37°C for 1 hour to induce LacI-GFP expression. Both longitudinal (B and C) and transverse (D and E) views of the lacO array are shown.

The disruption of interactions between aligned chromatids could also account for the changes in polytene chromosome structure caused by elevated dMi-2 expression. To test this hypothesis, we used the LacO/LacI-GFP system [31] to monitor the effect of dMi-2 over-expression on the structure of a specific locus by live analysis. In this system, a GFP-tagged Lac repressor (LacI-GFP) binds to tandem copies of Lac operator (LacO) sequences inserted at a single chromosomal location (Figure 7A). This assay has been successfully used to detect disruption of the normal parallel alignment of the chromatids in polytene chromosomes [32]. For this study, we used an insertion of a LacO transgene in an interband on the second chromosome and a transgene encoding LacI-GFP repressor under the control of the *Hsp83* promoter. Control *UAS-dMi-2^+^ 3-3/LacO LacI-GFP; UAS-dMi-2^+^ 15-1/da-GAL4 tubP-Gal80^ts^* individuals were reared at 18°C to the end of the third larval instar (240 hours) and heat-shocked for 60 minutes at 37°C to activate *LacI-GFP* expression. To over-express dMi-2, individuals of the same genotype were reared at 18°C for (9 days); shifted to 29°C for 27 hours to activate the *UAS-dMi-2^+^* transgenes; and heat-shocked for 60 minutes at 37°C to activate *LacI-GFP* expression. Polytene chromosomes of control larvae showed a compact band of GFP fluorescence signal perpendicular to the chromosome axis (Figure 7B and D). In larvae expressing elevated levels of dMi-2, however, the GFP signal was dispersed, with dots of LacI-GFP fluorescence scattered over a fivefold larger area (Figure 7C and E). This striking phenotype suggests that dMi-2 may play an unanticipated role in chromosome cohesion *in vivo*.

### dMi-2 destabilizes the association of cohesin with chromosomes

Cohesin is essential for sister chromatid cohesion in eukaryotic cells, and also plays important roles in gene expression, DNA repair, genome stability and chromatin organization [33], [34], [35], [36]. Four subunits of cohesin–Smc1, Smc3, Rad21 (Scc1/Mcd1) and stromalin (SA/Scc3/Stag2)–form a ring-like structure that encircles chromosomes from telophase until mitosis [33], [37]. Cohesin is loaded onto chromosomes by the kollerin complex containing the Nipped-B adherin protein, and is removed by the releasin complex and the separase protease to allow chromosome separation during anaphase [38].

**Figure 8:**
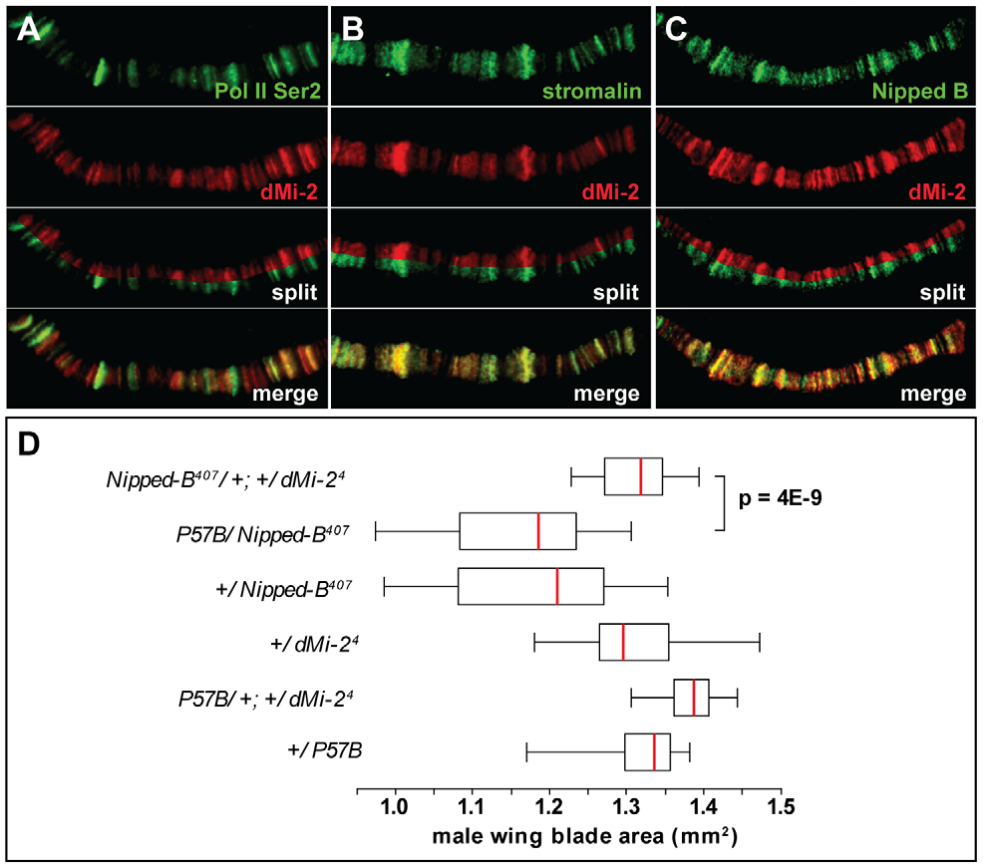
dMi-2 colocalizes with cohesin. (A–C) Magnified images of salivary gland polytene chromosomes stained with antibodies against dMi-2 (red) and Pol II Ser2 (A, green), stromalin (B, green) and Nipped B (C, green). Note the extensive overlap between the chromosomal distributions of the four proteins. (D) Reducing *dMi-2* gene dosage suppresses the small wing blade phenotype of individuals heterozygous for the *Nipped-B^407^* null allele. A minimum of twenty adult male wing blade areas was measured for each of the indicated genotypes, and the distributions of blade areas are presented as box-plots. For each genotype, the chromosome to the left of the separator (/) came from the male parent, and the chromosome to the right came from the female parent. The P57B chromosome is the wild-type chromosome in which the *Nipped-B^407^* mutation was induced by γ rays [39]. For the +/*Nipped-B^407^*, +/*dMi-2^4^* and +/P57B genotypes, the wild-type chromosomes came from an Oregon R male parent.

As a first step toward investigating potential interactions between dMi-2 and cohesin, we compared the distributions of dMi-2; the SA cohesin subunit, and the Nipped-B adherin on salivary gland polytene chromosomes of wild-type larvae. Interestingly, the distributions of both SA and Nipped-B were nearly identical to that of dMi-2 (Figure 8B and C). All three proteins were preferentially associated with less condensed, transcriptionally active regions of polytene chromosomes, as evidenced by the co-localization of dMi-2 and elongating RNA polymerase II (Pol II Ser2) (Figure 8A and Figure S5 A–F) [28].

We used genetic experiments to test the possibility that Nipped-B and dMi-2 functionally interact, as suggested by their co-localization. Heterozygous null *Nipped-B^407^* mutations reduce adult wing blade size relative to wild-type alleles, including the wild-type allele on the parental chromosome (P57B) on which *Nipped-B^407^* was induced (Figure 8D) [39]. The reduced wing size mirrors overall reduced body size, similar to that seen in mouse and human *Nipped-B* (*Nipbl*, *NIPBL*) heterozygotes (MG, DD unpublished) [40], [41], [42]. The heterozygous *dMi-2^4^* mutation suppresses the reduced wing size caused by heterozygous *Nipped-B^407^*. This genetic interaction suggests that dMi-2 antagonizes the function of the Nipped-B cohesin loading factor.

**Figure 9:**
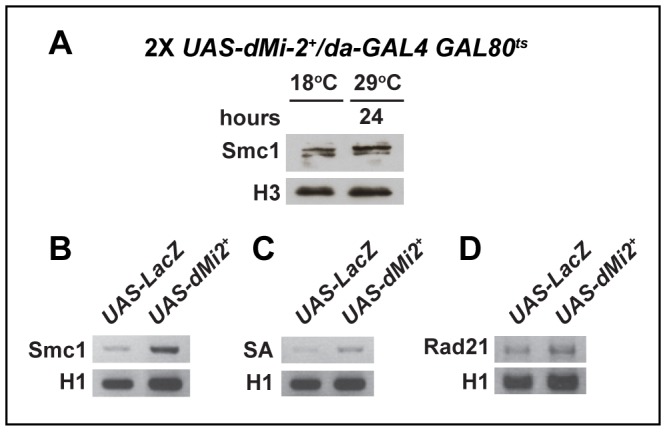
The over-expression of dMi-2 does not decrease cohesin levels. (A) Protein blot of salivary gland chromatin extracted from *UAS-dMi-2^+^ 3-3/+; UAS-dMi-2^+^ 15-1/da-GAL4 GAL80^ts^* individuals raised at 18°C until the late third-instar stage and then shifted to 29°C for 24 hours to induce *UAS-Mi-2^+^* expression. The blot was probed with antibodies against Smc1 and histone H3 as a control. (B, C and D) RT-PCR analysis of Smc1, SA, and Rad21 RNA levels in the salivary glands of *UAS-LacZ/+*; *da-GAL4/+* (*UAS-LacZ*) control larvae and *UAS-dMi-2^+^ 3-3/+*; *UAS-dMi-2^+^ 15-1/da-GAL4* (*UAS-Mi2^+^*) larvae raised at 29°C. Histone H1 RNA levels are shown as a control.

We examined whether the over-expression of dMi-2 alters polytene chromosome structure by altering the expression of cohesin subunits. The over-expression of dMi-2 for twenty-four hours using the GAL4-GAL80^ts^ system caused the dramatic decondensation of polytene chromosomes, but did not reduce the level of the Smc1 protein as assayed by western blotting (Figure 9A). The over-expression of dMi-2 slightly increased Smc1, SA, and Rad21 RNA in the salivary gland, as assayed by RT-PCR (Figure 9B–D). Although the cause of this increase is not known, it is clear that reduced cohesin expression is not responsible for dMi-2-dependent chromosome decondensation.

**Figure 10:**
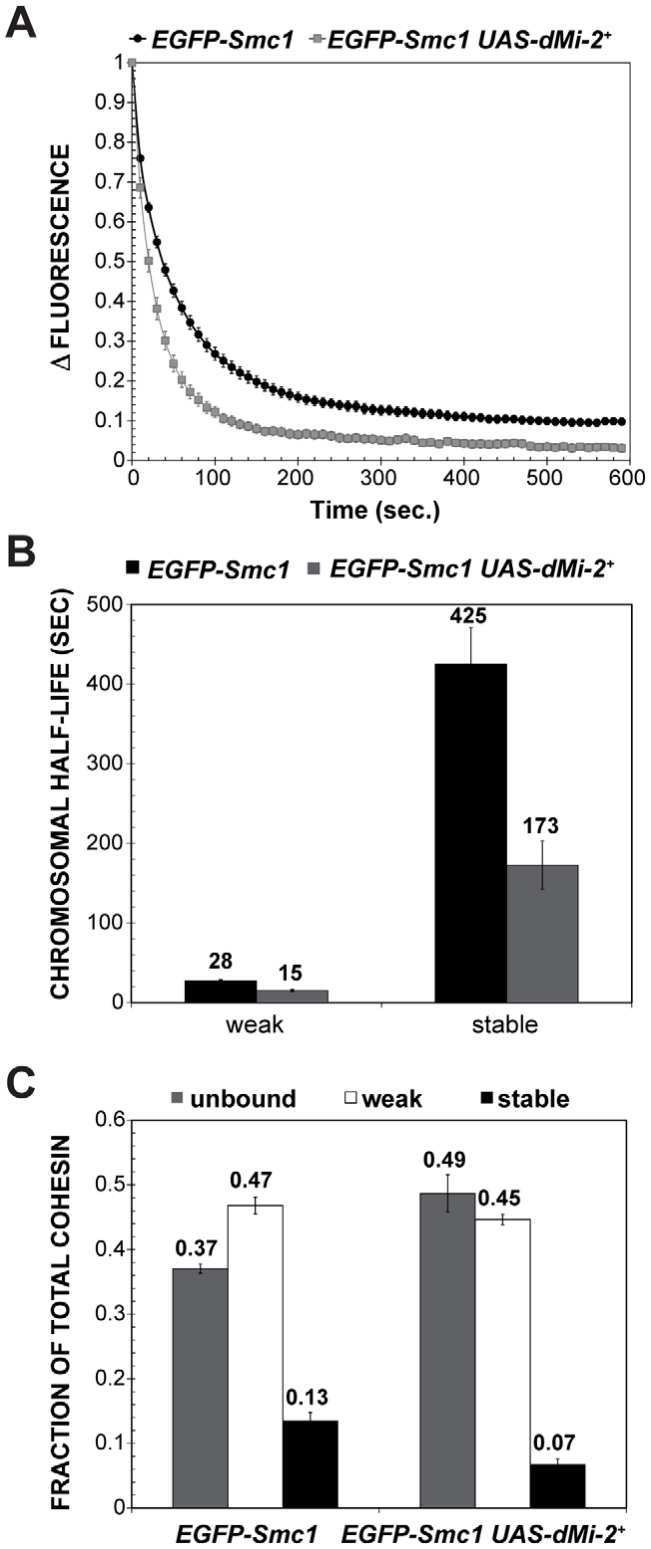
dMi-2 decreases the stability and extent of cohesin chromosome binding. (A) FRAP recovery curves for EGFP-Smc1 in wild-type salivary gland nuclei, and salivary gland nuclei in which *da-GAL4* drives the expression of two *UAS-dMi-2^+^* transgenes (*EGFP-Smc1 da-GAL4/UAS-dMi-2^+^ 3-3; UAS-dMi-2^+^ 15-1/+*) at 25°C. This induced a 1.6-fold average increase in nuclear volume (see text). EGFP-Smc1 was bleached in half the nucleus, and the curves show the decreasing difference in fluorescence intensity (Δ fluorescence) between the bleached and unbleached halves over time as the unbleached EGFP-Smc1 re-equilibrates throughout the nucleus. The curves shown are the average of at least 29 nuclei, and the error bars show the standard error of the means. (B) Curve-fitting distinguishes three cohesin fractions, unbound (determined by the loss of fluorescence in the unbleached half during bleaching, not shown), a weak binding fraction, and a stable binding fraction, presumed to be bound topologically. (C) Both the half-life of the stable binding cohesin (upper right panel) and total fraction of cohesin that binds in the stable mode were decreased by dMi-2 overexpression.

We next investigated whether dMi-2 antagonizes binding of cohesin to polytene chromosomes. Fluorescence recovery after photobleaching (FRAP) assays were used to analyze interactions between Smc1 and polytene chromosomes in third-instar larvae that express modest levels of EGFP-tagged Smc1 in addition to normal levels of endogenous Smc1 [43]. A previous study defined the existence of three dynamic forms of the cohesin complex in salivary glands: unbound, weakly bound and stably bound to chromosomes, with short and long equilibration half-lives for the weak and stable forms, respectively [43]. Moderate over-expression of dMi-2 with two transgenes at 25°C had a dramatic impact on the association of cohesin with polytene chromosomes. In *UAS-dMi-2^+^ 3-3/EGFP-Smc1; UAS-dMi-2^+^ 15-1/da-GAL4* larvae, there was a much faster equilibration between the bleached and the unbleached halves of the nucleus (Figure 10A), and the chromosomal half-life of the stable binding form decreased from 425 to 173 sec (Figure 10B). In addition, the fraction of stable EGFP-Smc1 binding decreased from 13% of the total to 7%, with a corresponding increase in the unbound fraction (Figure 10C). As expected, the nuclei with moderate dMi-2 overexpression were 1.6-fold larger in volume (9,784±627 µm^3^) on average than the control nuclei (5,986±337 µm^3^). Given the unusually long chromosomal residence time of stable cohesin, nuclear volume does not significantly affect the measured residence time through recapture of released cohesin [43]. A very large volume increase would be expected to increase recapture and cause an apparent increase in the measured chromosomal half-life instead of the observed decrease. These findings support the idea that dMi-2 promotes the dissociation of cohesin from polytene chromosomes. Reducing Nipped-B gene dosage similarly decreases the amount of stable cohesin, but in contrast to dMi-2 overexpression, does not alter its chromosomal residence time [43]. Thus it is unlikely that dMi-2 promotes cohesin dissociation by reducing Nipped-B activity.

### dMi-2 regulates higher-order chromatin structure in diploid cells

**Figure 11:**
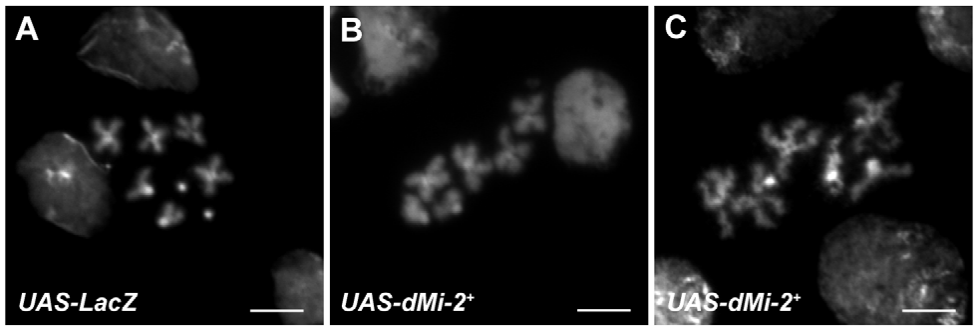
dMi-2 alters the structure of mitotic chromosomes. DAPI staining of mitotic chromosomes from the wing imaginal discs of *UAS-LacZ/+*; *da-GAL4/+* (*UAS-LacZ*) (**A**) and *UAS-dMi-2^+^ 3-3/+*; *UAS-dMi-2^+^ 3-3/da-GAL4* (*UAS-dMi-2^+^*) (B and C) third-instar larvae raised at 29°C. A,B and C scale bars are 5 µm.

To investigate whether dMi-2 affects an aspect of chromatin organization unique to polytene chromosomes, we examined whether its increased expression alters the structure of mitotic chromosomes in wing imaginal discs, a diploid tissue that is particularly well suited for cytological studies. The elevated expression of dMi-2 in *UAS-dMi-2^+^ 3-3/+; UAS-dMi-2^+^ 15-1/da-GAL4* larvae caused mitotic chromosomes to appear much less compact than normal (data not shown) without altering the mitotic index (1.4 vs. 1.3 in control larvae). To characterize this phenotype in more detail, we examined the metaphase chromosomes of imaginal discs treated with colchicine to induce mitotic arrest prior to fixation. In colchicine-treated wing discs of larvae over-expressing dMi-2, 20% of the metaphase chromosomes appeared disorganized and less compact than normal (compare figure 11B and C to A). Similar defects were not observed in colchicine-treated imaginal discs of control larvae. Roughly one-third of the abnormal metaphase chromosomes were also significantly longer than normal (third chromosome length = 3.3±0.4 µm (n = 43) vs. 5.4±1.0 µm (n = 63); compare figure 11C to A). These striking defects indicate that dMi-2 plays a general role in the regulation of higher-order chromatin structure in both diploid and polytene cells.

## Discussion

### dMi-2 promotes chromosome decondensation *in vivo*

Several lines of evidence suggest that the chromosome decondensation resulting from the increased expression of the dMi-2 protein reflects its normal function. The inducible transgene used in our studies rescued the recessive lethality of a *dMi-2* null allele indicating that it encodes a fully functional dMi-2 protein. Even a modest (two to threefold) increase in dMi-2 levels triggered chromosome decondensation; this effect was relatively rapid and was dependent on dMi-2 ATPase activity. The expression of a dominant-negative dMi-2 protein had the opposite effect on chromosome structure; the loss of dMi-2 function made polytene chromosomes appear more condensed than normal and blurred the distinction between bands and interbands. Taken together, these observations provide strong evidence that dMi-2 promotes chromosome decondensation *in vivo*.

### dMi-2 promotes local chromosome decondensation

HS puff formation exemplifies an unusually rapid and localized chromatin decondensation event. The precise mechanisms driving puff formation are not clear. HS gene activation is accompanied by nucleosome depletion, activation of PARP, extensive PARylation and the recruitment of topoisomerase, HSF, RNA polymerase II and several elongation factors as well as dMi-2 [18], [44], [45], [46]. Expression of dominant-negative dMi-2 protein reduces the transcriptional activation of HS genes but the underlying mechanisms have not been resolved [18]. Here, we have found that expression of a dominant negative dMi-2 protein leads to the formation of HS puffs with reduced size. This suggests that dMi-2 effects on chromosome structure are not always global. Instead, our results indicate that dMi-2 can be recruited to specific chromatin regions by environmental stimuli to contribute to the rapid local decondensation of chromatin. It is conceivable that the compromised decondensation of HS loci in dMi-2 mutants is one of the reasons for the observed loss in transcriptional output [18].

### dMi-2 and ISWI regulate higher-order chromatin structure via distinct mechanisms

To date, only one other *Drosophila* chromatin-remodeling factor, ISWI, has been implicated in the regulation of higher-order chromosome structure [7], [47], [48]. ISWI promotes the association of the H1 linker histone with interphase chromosomes [6], [8]. The loss of ISWI function leads to chromosome decondensation accompanied by the loss of histone H1, and the elimination of histone H1 causes chromosome defects that are remarkably similar to those resulting from dMi-2 over-expression [6], [8]. Although this similarity suggested that dMi-2 and ISWI have antagonistic effects on higher-order chromatin structure and histone H1 assembly, we failed to detect decreases in ISWI and histone H1 expression or histone H1 assembly following dMi-2 over-expression. dMi-2 and ISWI therefore appear to regulate distinct aspects of higher-order chromatin structure.

### dMi-2 regulates the dynamic association of cohesin with interphase chromosomes

Cohesin has been the topic of intensive study due to its critical role in sister chromatid cohesion during mitosis, and its roles in gene regulation and DNA repair. The complex forms a ring-like structure that encircles chromosomes beginning in telophase, and mediates sister chromatid cohesion upon DNA replication [33]. Cohesin binding is dynamic, but unusually stable compared to most DNA-binding proteins. Interphase cohesin is continuously loaded by the kollerin complex containing Nipped-B and released from chromosomes by the releasin complex containing Pds5 and Wapl [38], [43]. Our studies revealed an intriguing connection between dMi-2 and the cohesin complex, and argue that dMi-2 facilitates removal of cohesin from chromosomes during interphase. This activity is not restricted to situations in which dMi-2 is expressed at unusually high levels, since a twofold reduction in dMi-2 dosage counteracts the developmental consequences of reduced dosage of Nipped-B. Our findings add dMi-2 to the list of factors that regulate cohesin binding.

Cohesin regulates transcription by multiple mechanisms, including long-range interactions between insulators, enhancers and promoters via the formation of DNA loops, repression in collaboration with Polycomb proteins, and controlling transition of paused polymerase to elongation [34], [36], [49]. The observed suppression of a dominant *Nipped-B* mutant phenotype by reduced *dMi-2* gene dosage suggests that regulation of cohesin chromosome binding may be one mechanism by which dMi-2 controls gene expression.

The live analysis of a LacO array tagged with GFP in living cells is consistent with a potential role for dMi-2 in chromosome cohesion. The array is organized in a compact disc due to cohesion between precisely aligned chromatids. The over-expression of dMi-2 caused the LacO array to disperse into hundreds of discrete foci, presumably due to the disruption of interactions between sister chromatids. The over-expression of dMi-2 also disrupted the organization of mitotic chromosomes along their longitudinal axes, possibly by interfering with chromosomal interactions in *cis* that contribute to the organization of chromosome shape [50], [51].

Our findings show that dMi-2 plays unanticipated roles in both the regulation of higher-order chromosome structure and cohesin dynamics. Is there a causal relationship between the two activities? The sudden removal of cohesin in late larval development by targeted proteolysis does not dramatically alter polytene structure [52] and thus cohesin may not be critical for maintenance of polytene structure once fully established. However, genetic studies of *pds5* have revealed a role for both cohesin binding and sister chromatid cohesion in forming the normal structure of polytene chromosomes [53]. A *pds5* null allele and an allele encoding an N-terminally truncated protein alter polytene chromosome structure in distinctive ways, but in both cases the size and normal banding pattern are disrupted [53]. Taken together, the above considerations prevent us from concluding that dMi-2 promotes chromosome decondensation by destabilizing cohesin binding. However, because dMi-2 over-expression causes a large reduction in both the amount of stable cohesin and its chromosomal residence time, we can conclude that cohesin binding has been reduced, and that it is also likely that cohesion is affected.

The internal diameter of cohesin is ∼35 by 50 nm; it can therefore encircle only one 30 nm or two 10 nm chromatin fibers [54]. Interactions between cohesin complexes are thought to contribute to chromatid cohesion and presumably anchor chromatin loops to form “hubs” of high transcriptional activity [55], [56], [57]. The destabilization of cohesin binding therefore may be a secondary consequence of changes in chromatin structure catalyzed by dMi-2. Further work will be necessary to test this possibility and clarify the causal relationship, if any, between changes in chromosome structure and cohesin binding catalyzed by dMi-2.

It is intriguing that dMi-2, an antagonist of cohesin binding and well-characterized transcriptional repressor, co-localizes with cohesin at sites of active transcription. Although cohesin subunits and Nipped-B were not identified as stable subunits of dMi-2 containing complexes in cultured cells [13], [58], the extensive overlap between their chromosomal distributions suggests that chromatin structure and gene activity may be dependent on a fine balance of opposing dMi-2 and cohesin activities. Cohesin selectively binds and regulates active genes that have paused RNA polymerase, and can both positively and negatively regulate these genes by multiple mechanisms, including controlling the transition of paused polymerase to elongation [49]. It is possible that dMi-2 may also influence this transition by regulating cohesin binding and the chromatin structure at the pause sites. Intriguingly, mouse Mi-2ß and the NuRD complex bind active and poised gene promoters in thymocytes, and have both negative and positive effects on expression of these genes [59]. The Mi-2/NuRD complex regulates the expression of genes involved in lymphocyte differentiation [16], [60] and is also involved in stem cell renewal and determination [61], [62]. As in *Drosophila*, mammalian cohesin also regulates many genes critical for growth and development [34]. Our findings raise the interesting possibility that Mi-2 may regulate cellular differentiation in vertebrates by modulating chromosome condensation and cohesin activity.

## Materials and Methods

### *Drosophila* stocks and crosses

Flies were raised on cornmeal, agar, yeast and molasses medium, supplemented with methyl paraben and propionic acid. Mutations, chromosome aberrations, strains and abbreviations used in this study are described in Table S1 or Flybase (www.flybase.org) unless otherwise indicated. The amorphic *dMi-2^4^* allele is a frameshift mutation that blocks the production of functional dMi-2 protein [17]. The hypomorphic *dMi-2^f08104^* allele contains a PiggyBac transposon insertion in the first intron of the dMi-2 gene. The GAL4 and GAL4/GAL80^ts^ system and *sgs-GAL4*, *ey-GAL4* and *da-GAL4* drivers were used to activate the expression of *UAS* transgenes (Table S1) [20], [29], [63].

### Generation of Gal4-regulated transgenes

DNA fragments encoding C-terminal Flag-tagged dMi-2^+^ and dMi-2^Δ932-1158^ proteins were amplified from *dMi-2* cDNA clones [21] by PCR using the primers described in Table S2, subcloned into the *Not*I and *Xba*I sites of pVL1392, and transferred into the P-element transformation vector pUAST. To generate a GAL4-regulated transgene encoding N-terminal Flag-tagged KIS-L, we first subcloned the DNA fragment generated by the hybridization of two oligonucleotides shown in Table S2 into the *Eco*R1 and *Not*1 sites of pUAST to generate the pUAST-NFLAG vector. A 2.3 kb DNA fragment containing a *Not*1 site immediately upstream of the KIS-L start codon was amplified from the *kis2* cDNA [27] by PCR using the primers shown in Table S2. The resulting 2.3 kb *Not*1-*Eco*R1 fragment, together with the partially overlapping *kis2*, *kis30* and *kis40a* cDNA clones [27], was used to generate an approximately 17 kb *Not*1-*Kpn*1 fragment containing the entire KIS-L coding region and subcloned into the *Not*1 and *Kpn*1 sites of pUAST-NFLAG. To generate a GAL4-regulated transgene encoding C-terminal GFP-tagged histone H1, a fragment was amplified from *Drosophila* genomic DNA using the primers shown in Table S2, subcloned into the *Eco*R1 and *Xho*1 sites of pENTR1A and transferred into the P-element transformation vector pTWG [20], [64], [65], [66]. Transformants were generated using by *P*-element-mediated transformation using the *Df(1)w67c2* strain [67]. Homozygous viable transformants used in this study include *UAS-dMi-2^+^ 3-3*, *UAS-dMi-2^Δ932-1158^ 6-5*, *UAS-KIS-L^+^ 20-7* and *UAS-H1-GFP 2-1* on the second chromosome and *UAS-dMi-2^+^ 15-1* on the third chromosome (Table S1).

### Viability studies

To determine the viability of individuals bearing *dMi-2* mutations and transgenes, progeny of the crosses shown in Table 1 were scored. For each cross, ∼400 embryos were collected on grape juice/agar plates, transferred to vials and cultured at 25°C. Percent survival to each stage was calculated as the ratio between the number of individuals counted and the number of individuals expected for each genotype. The data for each of the crosses shown in Table 1 represent the average of at least two experiments.

### Analysis of fixed polytene and mitotic chromosomes

To analyze polytene chromosomes, salivary glands of late third-instar larvae were dissected in 0.7% NaCl and fixed in 1.85% formaldehyde/45% acetic acid as previously described [6]. Mitotic chromosomes of wing imaginal discs were examined as described previously for *Drosophila* neuroblasts [68].

To analyze the effect of dMi-2^Δ932-1158^ expression on chromosome structure, the progeny of *da-GAL4* females crossed to *UAS-dMi-2* ^Δ*932-1158*^ 6-5 males were shifted from 25°C to 29°C 48 hours after egg deposition. To analyze the effect of dMi-2 over-expression on chromosome structure, *da-GAL4* females, *Sgs3-GAL4* females or *ey-GAL4* females were crossed to *UAS-dMi-2^+^ 3-3* males, *UAS-dMi-2^+^ 15-1* males, or *UAS-dMi-2^+^ 3-3 UAS-dMi-2^+^ 15-1* males; the resulting progeny were raised at 29°C. To analyze the effect of Kismet over-expression on chromosome structure, the progeny of *ey-GAL4* females crossed to *UAS-KIS-L^+^ 20-7* males were raised at 25°C. To analyze the effect of dMi-2^K761R^ expression on puff formation, third-instar larvae from *da-GAL4* females crossed to *UAS-dMi-2^K761R^* males were shifted from 25°C to 37°C to induce the heat shock. Heat shock puff formation was induced by incubating two to four third-instar larvae in a 0.2 ml PCR tube for 20 minutes at 37°C; salivary glands were immediately dissected from the larvae in 0.7% NaCl pre-warmed to 37°C. Polytene chromosomes were fixed, squashed and stained with DAPI or antibodies [6].

Fixed preparations of polytene and mitotic chromosomes were analyzed with a Zeiss Axioskop 2 plus fluorescent microscope equipped with an AxioCam HRm CCD camera and images were acquired with a HCX PL APO CS 63.0×1.40 OIL objective and Axiovision 4.6.3 software [Zeiss]. For DNA quantification, images of DAPI stained chromosomes were captured using identical exposure times. Volocity 5.4 software (Perkin Elmer) was used to calculate the sum of pixel intensities within chromosomes boundaries and to calculate the Pearson's correlation coefficient for the co-localization of dMi-2 with Stromalin and Nipped-B.

### Live analysis of polytene chromosomes

Live analysis of polytene chromosomes was performed as previously described [8] in larvae bearing transgenes encoding GFP or mRFP1-tagged histone H2Av [69]. The level of histone H1 associated with chromosomes was analyzed using a transgene encoding GFP-tagged histone H1 (*UAS-H1-GFP*; Table S1) under the control of the *ey-GAL4* driver. Volocity software (release 5.4, Perkin Elmer) was used for three-dimensional reconstructions. For volume calculation we imaged sections of polytene nuclei every 0.5 µm. The change in chromatin compaction was established by calculating the ratio of nuclear volume to DNA content. The ratio for control samples was normalized to one.

### Antibodies and biochemical studies

Antibodies used in this study include rabbit polyclonal antibodies against dMi-2 (dMi-2C and dMi-2N) [70], *Drosophila* histone H1 (dH1 S72) [71], rabbit polyclonal *Drosophila* ISWI 196 [72], rabbit polyclonal *Drosophila* Rpd3 [70], *Drosophila* Smc1 [53], and histone H3 (Abcam, ab1791); a mouse monoclonal antibody against RNA PolIIo^ser2^ (H5, Covance); and guinea pig antibodies against Stromalin and Nipped B [53], [73]. The preparation of salivary gland chromatin extracts, SDS-PAGE and protein blotting were performed as previously described [6]. Baculoviral expression of dMi-2, dMi-2^Δ932-1158^, CAF1p55, dp66 and dMBD2/3 in Sf9 cells, extract preparation and anti-FLAG co-immunoprecipitation was carried out as described [13].

### GAL4 GAL80^ts^ system

*UAS-dMi-2^+^ 3-3/+; UAS-dMi-2^+^ 15-1/da-GAL4 Gal80^ts^* and *UAS-dMi-2* ^Δ932-1158^ 6-5/*+; +/da-GAL4 Gal80^ts^* individuals were shifted from 18°C to 29°C at the middle of the third larval instar to activate *UAS-dMi-2^+^* and *UAS-dMi-2* ^Δ932-1158^ expression, respectively. The time course of changes in chromosome structure following the shift to 29°C was monitored via both live analysis and the analysis of polytene chromosome squashes as described above. RNA levels in the salivary gland of *UAS-dMi-2^+^ 3-3/+; UAS-dMi-2^+^ 15-1/da-GAL4 Gal80^ts^* individuals were monitored by RT-PCR using primers specific to the RNAs encoded by Smc1, SA, Rad21, the endogenous *dMi-2^+^* gene or the *UAS-dMi-2^+^* transgene. Primers from the dMi-2 coding region were used to measure the overall increase in total *dMi-2* RNA levels (Table S2). RNA was isolated from salivary glands of ten *UAS-dMi-2^+^ 3-3/+; UAS-dMi-2^+^ 15-1/da-GAL4 Gal80^ts^* female larvae at specific times after the shift to 29°C and from ten control larvae maintained at 18°C using NucloSpin RNA XS (Clontech). cDNA was synthesized using the Superscript III First-Strand Synthesis System for RT-PCR kit (Invitrogen) and quantified with a nano-spectrophotometer using the NANODROP software. Identical amounts of cDNA were used for the PCR amplification of each sample.

### LacI-GFP/LacO and confocal microscopy

The progeny of *LacI-GFP LacO; da-GAL4 GAL80^ts^* females crossed to *UAS-dMi-2^+^ 3-3; UAS-dMi-2^+^ 15-1* males were allowed to develop to the third larval instar at 18°C (eight days). The expression of dMi-2 transgenes was activated by raising the temperature to 29°C for 27 hours, after which larvae were heat-shocked for 1–2 hours at 37°C to express *LacI-GFP*. After one hour of recovery at 29°C, salivary glands were dissected and polytene chromosomes were subjected to live analysis as described above. 0.2 µm sections of nuclei were imaged by confocal microscopy.

### Effects of *Nipped-B* and *dMi-2* mutations on wing size

Wings of adult progeny of crosses reared under uncrowded conditions at 25°C were mounted on microscope slides in Permount (Fisher Scientific) under coverslips. Digital images of the mounted wings were obtained using a 4X objective on a Nikon Microphot microscope (calibrated using a slide micrometer) and the blade areas measured using NIH ImageJ software (rsbweb.nih.gov/ij/). Statistical calculations and boxplots were generated using SSP software (economics files.pomona.edu/StatSite/ssp.html).

### Fluorescence recovery after photobleaching (FRAP)

The EGFP-Smc1 fusion protein and its functional characteristics were described previously [43]. Fluorescence recovery after photobleaching (FRAP) and data analysis was performed as previously described using salivary glands from late third-instar *UAS-dMi-2^+^ 3-3/Chip-EGFP-Smc1; UAS-dMi-2^+^15-1/da-GAL4* larvae raised at 25°C [43].

## Supporting Information

Figure S1 **dMi-2^Δ932-1158^ interacts with dNuRD subunits** *in vitro* **.** Subunits of the dNuRD complex were expressed in Sf9 cells either alone (left panels), with flag-tagged dMi-2^+^ (middle panels) or with flag-tagged dMi-2^Δ932-1158^ (right panels) using recombinant baculoviruses. Whole cell extracts were immunoprecipitated with flag-beads. Immunoprecipitates were analysed by protein blotting using the antibodies indicated on the left. IN = input (extract); IP = immunoprecipitate.
(TIF)

Figure S2 **The over-expression of dMi-2^+^ alters chromosome structure.** (A–B) Salivary gland polytene chromosome squashes of *UAS-LacZ/+; Sgs-GAL4/+* (UAS-LacZ) control larvae (A) and *UAS-dMi-2^+^ 3-3/+; UAS-dMi-2^+^15-1/Sgs-GAL4* (UAS-dMi-2^+^) larvae (B) stained with DAPI. The over-expression of dMi-2 in late third-instar larvae under the control of the Sgs3-GAL4 driver increases the size of polytene chromosomes and disrupts their banding pattern (compare B to A). A and B scale bars are 10 µm.
(TIF)

Figure S3 **The over-expression of dMi-2^+^ rapidly induces changes in chromosome structure.** (A–C) DAPI stained salivary gland polytene chromosome squashes of *UAS-dMi-2^+^ 3-3/+; UAS-dMi-2^+^ 15-1/da-GAL4 GAL80^ts^* (UAS-dMi-2^+^) two (A) and ten (B) hours following the shift from 18 to 29°C to induce UAS-dMi-2^+^ expression. (C) *UAS-LacZ/+; da-GAL4 GAL80^ts^/+* (UAS-LacZ) used as a control. Chromosome decondensation and disruption of the banding pattern was evident within 10 hours. A, B and C scale bars are 10 µm.
(TIF)

Figure S4 **Effect of dMi-2 on histone H1 and ISWI expression in the salivary glands of third-instar larvae.** (A) A Western blot of salivary gland proteins extracted from the salivary glands of larvae over-expressing wild-type dMi-2 for 24 hours was probed with antibodies against ISWI and histone H3 as a control. (B) A Western blot of proteins extracted from the salivary glands of larvae expressing dominant-negative dMi-2 for 24 hours was probed with antibodies against histone H1 and histone H3 as a control. The ratio of the histone H1 and H3 signals are indicated. Note that the over-expression of dMi-2 does not alter ISWI levels. The expression of dominant-negative dMi-2 leads to a slight decrease in histone H1 levels.
(TIF)

Figure S5 **Cohesin colocalizes with dMi-2 and RNA Pol II.** (A) Upper panel, magnified image of a portion of a salivary gland polytene chromosome stained with an antibody against dMi-2 (red) and DAPI (blue). Lower panel, linear plot profile showing that dMi-2 is associated primarily with less condensed regions.. The dashed lines indicate the corresponding location of dMi-2 bands in the plot. (B–D) Merged images of wild-type polytene chromosomes showing the colocalization of dMi-2 (red) with Pol II Ser2 (B, green), with stromalin (C, green) and with Nipped B (D, green). (E–G) Pairwise scatter plot of the intensities of overlapping pixels of dMi-2 and Pol II Ser2 (E), stromalin (F) and Nipped B (G) staining in the images shown in panels B, C and D, respectively. dMi-2 is represented by red dots while Pol II Ser2, Nipped B and stromalin are shown as green dots. The color of the dots indicates the level of colocalization, with yellow indicating perfect overlap. B, C and D scale bar is 10 µm.
(TIF)

Table S1 **The full genotypes of the stocks used in this study, their corresponding abbreviations and their sources are indicated.**
(DOC)

Table S2 **List of forward and reverse oligonucleotide primers used for the construction of vectors and transgenes and the quantification of RNA levels by RT-PCR.** The templates used, primer names, and corresponding sequences are shown.
(DOC)
