# Supplementary figures and images for: The Drosophila Mi-2 Chromatin-Remodeling Factor Regulates Higher-Order Chromatin Structure and Cohesin Dynamics In Vivo
Source: PLoS Genet. 2012 Aug 9;8(8):e1002878. doi: 10.1371/journal.pgen.1002878 (PMC3415455; doi:10.1371/journal.pgen.1002878)

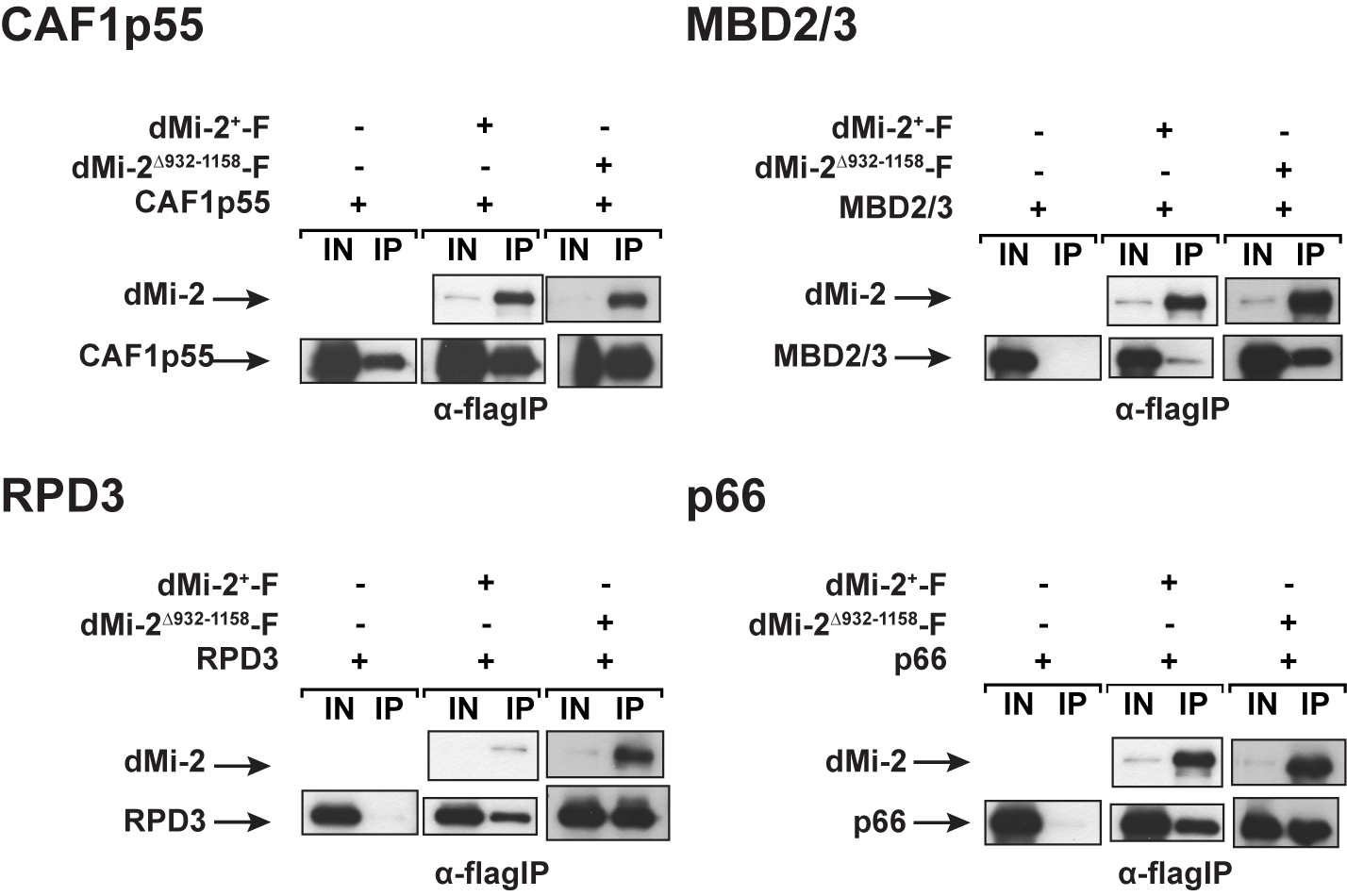

Supplement: Figure S1 — dMi-2Δ932-1158 interacts with dNuRD subunits in vitro . Subunits of the dNuRD complex were expressed in Sf9 cells either alone (left panels), with flag-tagged dMi-2+ (middle panels) or with flag-tagged dMi-2Δ932-1158 (right panels) using recombinant baculoviruses. Whole cell extracts were immunoprecipitated with flag-beads. Immunoprecipitates were analysed by protein blotting using the antibodies indicated on the left. IN = input (extract); IP = immunoprecipitate. (TIF) [file pgen.1002878.s001.tif]

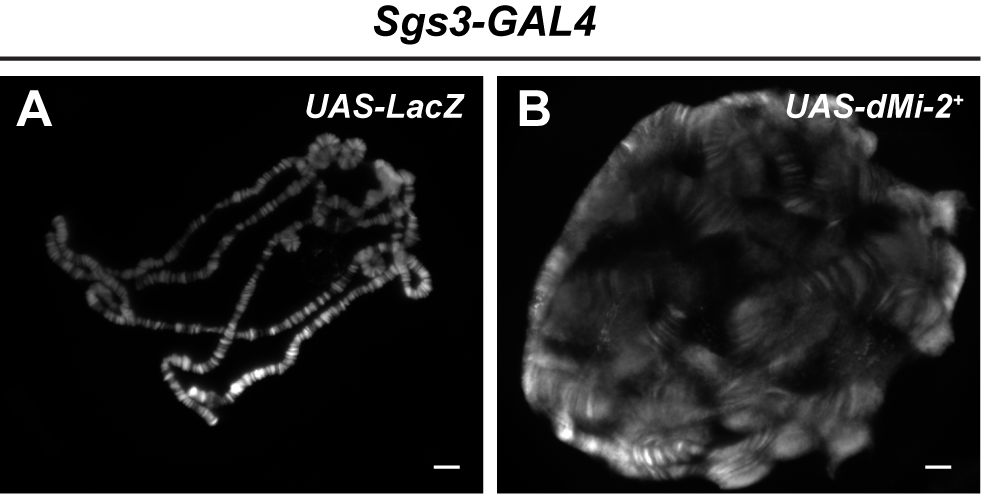

Supplement: Figure S2 — The over-expression of dMi-2+ alters chromosome structure. (A–B) Salivary gland polytene chromosome squashes of UAS-LacZ/+; Sgs-GAL4/+ (UAS-LacZ) control larvae (A) and UAS-dMi-2+ 3-3/+; UAS-dMi-2+15-1/Sgs-GAL4 (UAS-dMi-2+) larvae (B) stained with DAPI. The over-expression of dMi-2 in late third-instar larvae under the control of the Sgs3-GAL4 driver increases the size of polytene chromosomes and disrupts their banding pattern (compare B to A). A and B scale bars are 10 µm. (TIF) [file pgen.1002878.s002.tif]

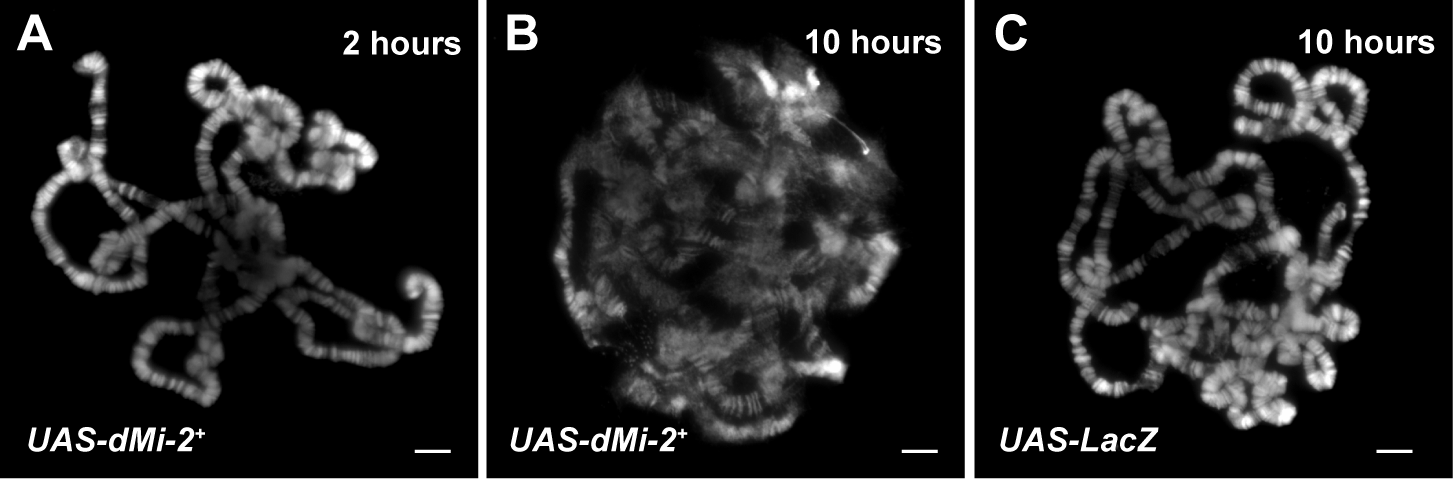

Supplement: Figure S3 — The over-expression of dMi-2+ rapidly induces changes in chromosome structure. (A–C) DAPI stained salivary gland polytene chromosome squashes of UAS-dMi-2+ 3-3/+; UAS-dMi-2+ 15-1/da-GAL4 GAL80ts (UAS-dMi-2+) two (A) and ten (B) hours following the shift from 18 to 29°C to induce UAS-dMi-2+ expression. (C) UAS-LacZ/+; da-GAL4 GAL80ts/+ (UAS-LacZ) used as a control. Chromosome decondensation and disruption of the banding pattern was evident within 10 hours. A, B and C scale bars are 10 µm. (TIF) [file pgen.1002878.s003.tif]

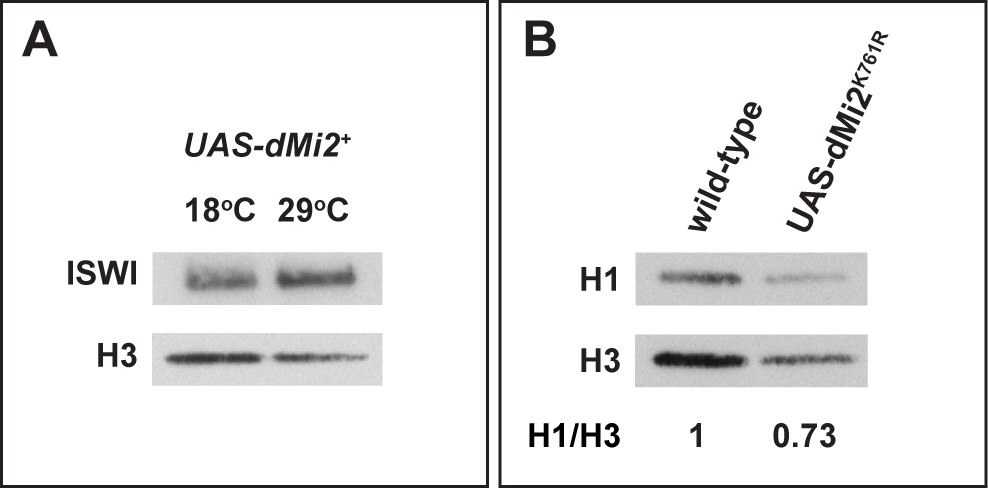

Supplement: Figure S4 — Effect of dMi-2 on histone H1 and ISWI expression in the salivary glands of third-instar larvae. (A) A Western blot of salivary gland proteins extracted from the salivary glands of larvae over-expressing wild-type dMi-2 for 24 hours was probed with antibodies against ISWI and histone H3 as a control. (B) A Western blot of proteins extracted from the salivary glands of larvae expressing dominant-negative dMi-2 for 24 hours was probed with antibodies against histone H1 and histone H3 as a control. The ratio of the histone H1 and H3 signals are indicated. Note that the over-expression of dMi-2 does not alter ISWI levels. The expression of dominant-negative dMi-2 leads to a slight decrease in histone H1 levels. (TIF) [file pgen.1002878.s004.tif]

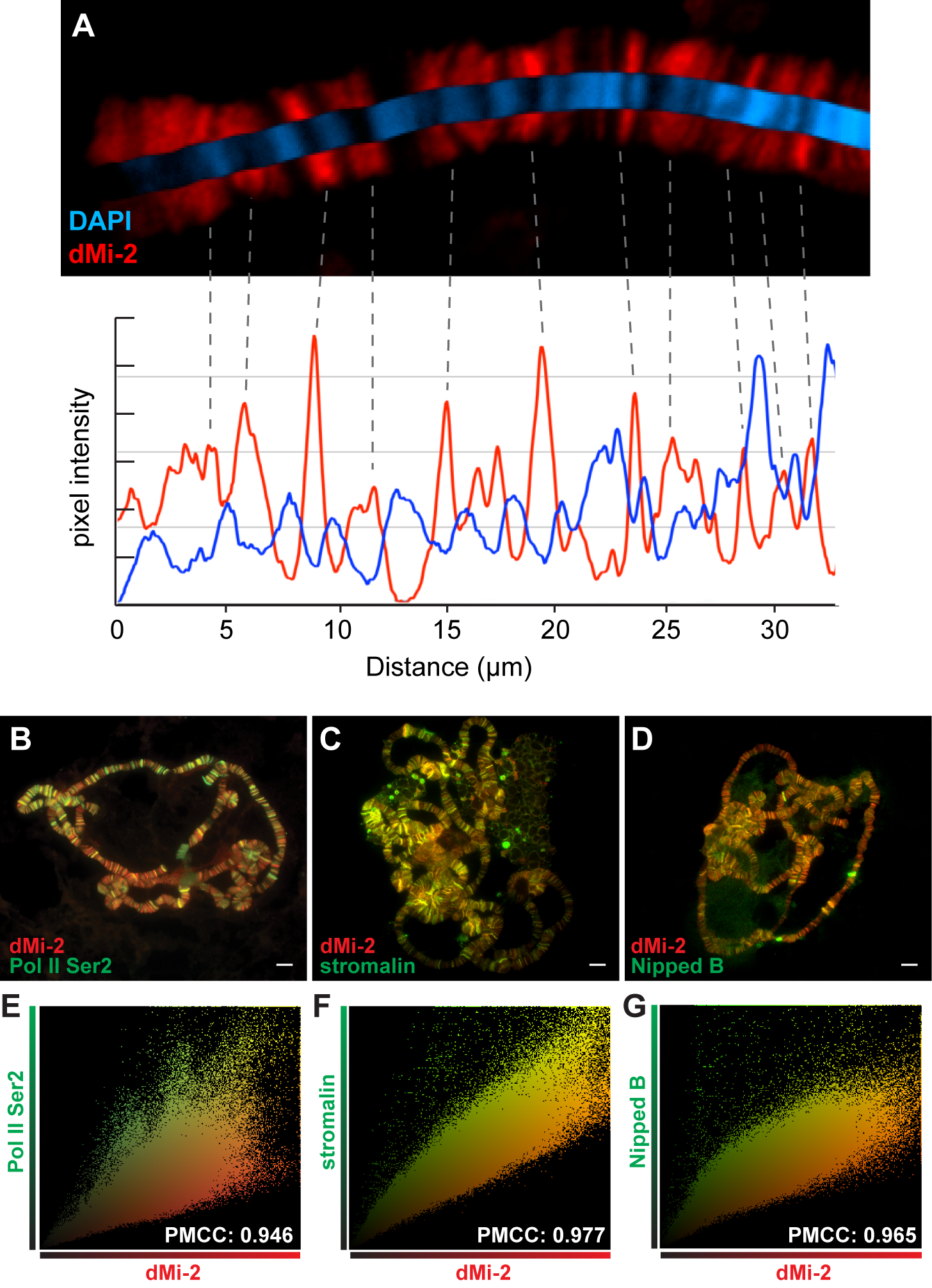

Supplement: Figure S5 — Cohesin colocalizes with dMi-2 and RNA Pol II. (A) Upper panel, magnified image of a portion of a salivary gland polytene chromosome stained with an antibody against dMi-2 (red) and DAPI (blue). Lower panel, linear plot profile showing that dMi-2 is associated primarily with less condensed regions.. The dashed lines indicate the corresponding location of dMi-2 bands in the plot. (B–D) Merged images of wild-type polytene chromosomes showing the colocalization of dMi-2 (red) with Pol II Ser2 (B, green), with stromalin (C, green) and with Nipped B (D, green). (E–G) Pairwise scatter plot of the intensities of overlapping pixels of dMi-2 and Pol II Ser2 (E), stromalin (F) and Nipped B (G) staining in the images shown in panels B, C and D, respectively. dMi-2 is represented by red dots while Pol II Ser2, Nipped B and stromalin are shown as green dots. The color of the dots indicates the level of colocalization, with yellow indicating perfect overlap. B, C and D scale bar is 10 µm. (TIF) [file pgen.1002878.s005.tif]
