# Supplementary material for: The Drosophila Mi-2 Chromatin-Remodeling Factor Regulates Higher-Order Chromatin Structure and Cohesin Dynamics In Vivo
Source: PLoS Genet. 2012 Aug 9;8(8):e1002878. doi: 10.1371/journal.pgen.1002878 (PMC3415455; doi:10.1371/journal.pgen.1002878)
Supplement: Table S1 — The full genotypes of the stocks used in this study, their corresponding abbreviations and their sources are indicated. (DOC) [file pgen.1002878.s006.doc]

Table S1

|  | **Abbreviation** | **Source** |
| --- | --- | --- |
| **Gal4 drivers** |  |  |
| P[da-GAL4.w^-^]3 | da-GAL4 | Bloomington Stock Center |
| P[w^+^, Sgs3-GAL4.PD]TP1 | Sgs-GAL4 | Bloomington Stock Center |
| P[w^+^,ey-GAL4.Exel]2 | ey-GAL4 | Bloomington Stock Center |
| **Mutations and transgenes** |  |  |
| dMi-2^4^ | dMi-2^4^ | Juerg Müller (EMBL) |
| [PBac[WH]dMi-2^f08103^](http://flybase.org/reports/FBti0053840.html) | dMi-2^f08103^ | Exelixis/Harvard Medical School |
| P[w^+^, UAS-dMi-2^∆932-1158^]6-5 | UAS-dMi-2^∆932-1158^ 6-5 | Present study |
| P[w^+^, UAS-dMi-2^K761R^] | UAS-dMi-2^K761R^ |  |
| P[w^+^, UAS-dMi-2^+^]3-3 | UAS-dMi-2^+^ 3-3 | Present study |
| P[w^+^, UAS-dMi-2^+^]15-1 | UAS-dMi-2^+^ 15-1 | Present study |
| P[w^+^,His2Av-mRFP1]III.1 | H2Av-mRFP | Bloomington Stock Center |
| P[w^+^,His2Av^T:Avic\GFP-S65T^]62A | H2Av-GFP | Bloomington Stock Center |
| P[w^+^, tubP-GAL80^ts^] | tubP-Gal80^ts^ | Bloomington Stock Center |
| M1.1(LacO array at 60F) 6(4.1) HS83-LacI-GFP | LacI-GFP, LacO | Giovanni Bosco (University of Arizona) |
| P[w^+^,UAS-LacZ] | UAS-LacZ | Bloomington Stock Center |
| P[w^+^, UAS-KIS-L^+^]20-7 | UAS-KIS-L^+^ | Present study |
| P[w^+^, UAS-His1-GFP]2-1 | H1-GFP | Present study |
| EGFP-Smc1 | EGFP-Smc1 |  |

**SUPPLEMENTAL REFERENCES**

1. Murawska M, Hassler M, Renkawitz-Pohl R, Ladurner A, Brehm A (2011) Stress-induced PARP activation mediates recruitment of Drosophila Mi-2 to promote heat shock gene expression. PLoS Genet 7: e1002206.

2. Gause M, Misulovin Z, Bilyeu A, Dorsett D (2010) Dosage-sensitive regulation of cohesin chromosome binding and dynamics by Nipped-B, Pds5, and Wapl. Mol Cell Biol 30: 4940-4951.
