# Supplementary material for: The Drosophila Mi-2 Chromatin-Remodeling Factor Regulates Higher-Order Chromatin Structure and Cohesin Dynamics In Vivo
Source: PLoS Genet. 2012 Aug 9;8(8):e1002878. doi: 10.1371/journal.pgen.1002878 (PMC3415455; doi:10.1371/journal.pgen.1002878)
Supplement: Table S2 — List of forward and reverse oligonucleotide primers used for the construction of vectors and transgenes and the quantification of RNA levels by RT-PCR. The templates used, primer names, and corresponding sequences are shown. (DOC) [file pgen.1002878.s007.doc]

**Table S2**

| Template | Oligonucleotide | Sequence (5’ to 3’) |
| --- | --- | --- |
| **Construction of UAS-H1-GFP transgene** | | |
| H1 | H1-GFP C-ter 5’ | ACCGAATTCGCATGTCTGATTCTGCAGTTGCA |
| H1 | H1-GFP C-ter 3’ | TATCTCGAGTGCTTTTTGGCAGCCGTAGT |
| **Construction of pUAST NFLAG vector** | | |
| - | NFLAG 5’ | AATTCAAAATGGACTACAAGGACGACGATGACAAGTGC |
| - | NFLAG 3’ | GGCCGCACTTGTCATCGTCGTCCTTGTAGTCCATTTTG |
| **Insertion of Not1 site upstream of Kis start codon** | | |
| Kis | Kis-Not1-AUG 5’ | CTGCGGCCGCATGGATGGAAATGC |
| Kis | Kis-Not1-AUG 3’ | GCGAATTCGTGTTGTCCATGAGCAACTGTT |
| **Primers sets used for RT-PCR** | | |
| dMi-2 | dMi-2 N-ter 5’ ^a)^ | ATGAAGAGCGATGTGTCCCG |
| dMi-2 | dMi-2 UTR ^b)^ | TTCGCCAAGTGTCAAACG |
| dMi-2 | dMi-2 N-ter 3’ ^c)^ | TCGTGAATGCTGGCAATG |
| Flag | Flag-3’ ^d)^ | TGTCATCGTCATCCTTGTAGTC |
| ISWI | ISWI-5’ | GGTCAAAAGAAGCAAGTGCGCGTT |
| ISWI | ISWI-3’ | GGGGTGGGAAGAACTGAAAGTC |
| Stromalin | SA-5’ | TGACCGTTTGGACTCGCTTATG |
| Stromalin | SA-3’ | TCGCCTTCTCATCTTGAATAGCC |
| Smc1 | Smc1-5’ | GGAAGTTTACGAGCGATTTGGAAGTG |
| Smc1 | Smc1-3’ | TAGCGTTTATGCGTCGGCTGAC |
| Rad21 | Rad21-5’ | ATGACTTTGATACAGCCCTGCCCG |
| Rad21 | Rad21-3’ | CGACACCTCCAGTTTCTTTCCAATAC |
| H1 | H1-5’ | AGCCTCTAAGAAGATTGGTGTCTCC |
| H1 | H1-3’ | GCCTTTGACGCTTTCGCTACTAC |

^a)^ 5’ primer for dMi-2 endogenous, dMi-2 induced and total dMi-2

^b)^ 3’ primer for dMi-2 endogenous

^c)^ 3’ primer for dMi-2 endogenous and induced

^d)^ 3’ primer for dMi-2 induced
